# LMOD1, an oncogene associated with Lauren classification, regulates the metastasis of gastric cancer cells through the FAK-AKT/mTOR pathway

**DOI:** 10.1186/s12885-022-09541-0

**Published:** 2022-04-30

**Authors:** Yuen Tan, Qingchuan Chen, Siwei Pan, Wen An, Huimian Xu, Yao Xing, Jianjun Zhang

**Affiliations:** 1 Department of Gastric Surgery, Liaoning Cancer Hospital and Institute, Cancer Hospital of China Medical University, No 44 of Xiaoheyan Road, Dadong District, Shenyang, 110042 China; 2 Department of Surgical Oncology, First Affiliated Hospital of China Medical University, 155 Nanjing North Street, Heping District, Shenyang, 110001 China; 3 Department of Cell Biology, Key Laboratory of Cell Biology of Ministry of Public Health, and Key Laboratory of Medical Cell Biology of Ministry of Education, China Medical University, No. 77, Puhe Road, Shenyang North New Area, Shenyang, 110122 Liaoning China

**Keywords:** Lauren classification, WGCNA, LMOD1, FAK-Akt/mTOR, Peritoneal metastasis

## Abstract

**Background:**

The Lauren classification of gastric tumors strongly correlates with prognosis. The purpose of this study was to explore the specific molecular mechanism of Lauren classification of gastric cancer and provide a possible theoretical basis for the treatment of gastric cancer.

**Methods:**

We standardized the gene expression data of five Gene Expression Omnibus gastric cancer databases and constructed a Weighted Co-expression Network Analysis (WGCNA) model based on clinicopathological information. The overall survival (OS) and disease-free survival (DFS) curves were extracted from the Cancer Genome Atlas (TCGA) and GSE62254 databases. Western blotting was used to measure protein expression in cells and tissues. Scratch and transwell experiments were used to test the migration ability of tumor cells. Immunohistochemistry was used to measure tissue protein expression in clinical tissue samples to correlate to survival data.

**Results:**

The WGCNA model demonstrated that blue cyan was highly correlated with the Lauren classification of the tumor (r = 0.24, *P* = 7 × 10^16^). A protein-protein interaction network was used to visualize the genes in the blue cyan module. The OS and PFS TCGA analysis revealed that LMOD1 was a gene of interest. The proportion of diffuse gastric cancer patients with high expression of LMOD1 was significantly higher than that of intestinal type patients. LMOD1 promoted the migration of gastric cancer cells by regulating the FAK-Akt/mTOR pathway in vitro. Additionally, a Gene Set Enrichment Analysis using the TCGA and GSE62254 databases, and western blot data, showed that LMOD1 could promote an epithelial-mesenchymal transition (EMT), thus potentially affecting the occurrence of peritoneal metastasis of gastric cancer. Immunohistochemistry showed that LMOD1 was highly expressed in cancer tissues, and the prognosis of patients with high LMOD1 expression was poor.

**Conclusion:**

LMOD1 is an oncogene associated with diffuse gastric cancer and can affect the occurrence and development of EMT by regulating the FAK-Akt/mTOR pathway. LMOD1 can therefore promote peritoneal metastasis of gastric cancer cells and can be used as a novel therapeutic target for gastric cancer.

**Supplementary Information:**

The online version contains supplementary material available at 10.1186/s12885-022-09541-0.

## Introduction

Gastric cancer is the fifth most common type of cancer, and fourth leading cause of cancer-related death [1]. The occurrence and development of gastric cancer is a multi-gene and multi-stage process, including the proliferation and metastasis of tumor cells [2, 3]. In recent years, there has been great progress in the treatment of gastric cancer. The application of targeted therapy and immunotherapy has greatly improved the survival rate of advanced gastric cancer, but not all patients respond to treatment, especially for patients with peritoneal metastasis [4, 5]. Peritoneal metastasis is one of the main factors affecting the prognosis and quality of life of gastric cancer patients. Therefore, it is of great significance to identify the key genes affecting the progression of peritoneal metastasis of gastric cancer and target them in clinical treatment to improve the prognosis of patients.

The classification systems used with gastric cancer include Lauren classification, Borrmann classification, and the serosal classification. The Lauren classification divides tumors into an intestinal type, diffuse type and mixed type [6]. The survival time of patients with diffuse gastric cancer is significantly shorter than that of patients with intestinal gastric cancer, where the 5-year survival rate for the intestinal type is 57.7%, and the 5-year survival rate for the diffuse type is only 45.6% [7]. Peritoneal metastasis is reported in 80% of diffuse gastric cancer patients, which is much higher than for intestinal gastric cancer patients [8]. Gene therapy for Lauren typing may greatly reduce peritoneal metastasis of gastric cancer and could be a potential approach for the treatment of gastric cancer.

Weighted Gene Co-expression Network Analysis (WGCNA) is an efficient and accurate data mining method [9]. By inducing genes with similar expression changes, we can identify co-expression modules and explore the relationship between gene network and phenotype. An increasing number of scholars use WGCNA in gene mining of various tumors, which greatly improves the efficiency of basic research [10, 11]. If the expression of genes in different patient tissues is similar, these genes can be thought of as functionally related. Genomes with similar expression profiles form a module. Leiomodin 1 (LMOD1) was initially identified as 64 kDa autoantigen in a serum expression screen in Hashimoto’s thyroiditis. The gene transcript was found to be expressed in all tissues tested, with the highest levels being in the thyroid, eye muscle, and skeletal muscle [12]. In recent years, immunohistochemistry and western blotting for LMOD1 have demonstrated its highly restricted expression pattern in smooth muscle cell lines, such as adult aorta and bladder, suggesting that LMOD1 may be related to the phenotype of smooth muscle differentiation.

In this study, we constructed a WGCNA analysis model by combing the information on gastric cancer patients from Gene Expression Omnibus (GEO) databases, and selected LMOD1 as our research gene target for the modules highly related to the Lauren classification data. Western blotting results and functional tests showed that LMOD1 can induce gastric cancer cell migration by regulating the FAK-Akt/mTOR pathway and can promote the occurrence and development of gastric cancer peritoneal metastasis by affecting the epithelial-mesenchymal transition (EMT) of gastric cancer cells.

## Materials and methods

### Cell culture

The GES-1, SGC-7901, MGC-803, HGC-27 and AGS cell lines were purchased from the Cell Culture Collection of the Chinese Academy of Sciences (Shanghai, China). The Sv5 cell line (human peritoneal mesothelial cell, HPMC) was kindly provided by You-Ming Peng (Second Hospital of Zhongnan University, China). DMEM and RPMI-1640 medium were used for cell culture (Biological Industries, Israel), and the proportion of serum in cell culture medium was 10% (Biological Industries, Israel).

### Data sources and data preprocessing

We searched “stomach neoplasms” [MeSH terms] or gastric cancer [All Fields]) and Lauren [All Fields] in the public database, and identified five datasets (GSE15460, GSE29272, GSE62254, GSE13861 and GSE26253) containing a total of 1001 gastric cancer patients with complete clinical information. The data from GSE29272 and GSE62254 were normalized by robust multichip average (RMA). The Illumina data (GSE13861, GSE26253) were normalized by quantity normalization and log2 transformation.

### Construction of the co-expression network

We extracted and screened gene expression data from 8135 genes from five GEO databases, analyzed the expression of each gene in 1001 patient samples by ANOVA, and took the top 25% of genes (2035) for subsequent WGCNA analysis. We used the WGCNA package for the WGCNA analysis, and the cluster analysis was performed on the expression data, and clinical information of 1001 patients to measure their distribution, and eliminate abnormal values. After the modules were obtained, the modules with similarity greater than 0.75 were merged to obtain 12 co-expression modules.

### Gene set enrichment analysis

For the Gene Set Enrichment Analysis (GSEA), gene expression in the TCGA and GSE62254 datasets were divided into two groups according to the high and low expression of LOMD1. Gene pathway enrichment was analyzed by GSEA software (4.1.0) [13].

### Vector construction and transfection

The silenced GFP-LMOD1 Lentivirus was purchased from Beijing Syngenesis (China). The type of GFP-LMOD1 virus was PHS-ASR-0921(pLV-hU6-shRNA-hef1a-mNeogreen-P2A-Puro) and lentivirus target was GGAGATGTCCATGGATGAAAG. Puromycin (5 μg/ml) was used to screen viral-transduced stable cell lines. The LMOD1 overexpression plasmid was purchased from Genechem (Shanghai, China). The nucleotide sequence for the LMOD1 overexpression plasmid is shown in the Supplement word. Higene was used as the transfection reagent for plasmid transfection, and G418 was used for stable transformation screening.

### Western blot

Western blotting was performed as previously described [14, 15]. Briefly, cells were collected, lysed, and centrifuged. SDS-PAGE gel was used to separate equal amounts of proteins. Proteins were then transferred added to PVDF membranes (Millipore). The membranes were blocked in 5% non-fat milk and incubated with primary antibodies at 4 °C overnight. After 1 h incubation with secondary antibodies, the membranes were developed with a chemiluminescence solution. The primary antibodies used were: LMOD1 (Accept), FAK (Proteintech), AKT (CST), p-AKT (CST), mTOR (Proteintech), p-mTOR (Proteintech), E-cadherin (CST), N-cadherin (CST), vimentin (CST), and actin (Proteintech). We cut the membrane to facilitate the better hybridization of antibodies and avoid the interaction of antibodies. The original pictures were attached to the Supplementary figure_ Original Western blots.

### Real-time quantitative PCR analysis

After total RNA was extracted from the cells, 1 μg of RNA was added into collection tubes, and an appropriate amount of reverse transcription reagent (Takara, Japan) was added to obtain the cDNA product for quantitative analysis. SYBR dye (Takara, Japan) was added to the reactions, and the number of cycles on the thermal cycler was set to 45. GAPDH was used as a reference gene. The primer sequences were: LMOD1 5′- GAAGAACTCCCGTGACCAGCTA-3′ (sense) and 5′-AGCCTGGTCCTACTGAAGCAGT-3′ (antisense), GAPDH 5′- GTCTCCTCTGACTTCAACAGCG-3′ (sense) and 5′- ACCACCCTGTTGCTGTAGCCAA-3′ (antisense).

### Wound-healing assay

Cells were trypsinized, resuspended, and 5 × 10^5^ cells were evenly seeded in a 6-well plate. After the cells reached confluency a 200 μL pipette tip was used to make a scratch in the cell layer. Wells were washed twice with PBS and 2 mL of serum-free medium was added. An image was acquired at time zero. After 24 h, wells were washed twice with PBS and imaged.

### Migration assay

Cells were trypsinized and resuspended in serum-free medium. A total of 3 × 10^5^ / 200 μL medium were added into a Transwell chamber (Corning, USA), and 600 μL serum-containing medium was added into the lower chamber. After 18-22 h, the chambers were moved and washed twice with PBS. Chambers were immersed in 95% alcohol for 20 min, washed in PBS, and 0.4% trypan blue solution was added for 15 min. After washing with PBS, the inner surface of the chamber membrane was wiped with a cotton swab. Images were acquired.

### Adhesion and invasion assay

Adhesion and invasion experiments relating to peritoneal metastasis were carried out according to previous experiments [15]. For the adhesion experiment, HPMCs cocultured with gastric cancer cells were plated in 12 well plates. 2 × 10^5^ GC cells incubated in 5 μmol /L Calcein-AM (Sigma) for 1 h, then added to 12 well plates and cultured for 2 h. The plates were washed with PBS to remove suspended GC cells. The number of GC cells was counted under a fluorescence microscope. For the invasion experiment, HPMCs cocultured with GC cells were plated in a chamber (8-μm pore size; Corning) containing matrigel for 4-6 h. 5 × 10^5^ GC cells resuspended in 100 μl serum-free medium were added to the HPMC layer. 600 μl of medium containing 10% FBS was added to the lower chamber. After 28-32 h, we removed the upper chamber for fixation and staining, and counted the number of cells under the microscope.

### Samples and patients

We collected 102 cases of gastric cancer tissues and 40 cases of paracancerous tissues in our department from 2011 to 2012. These tissues were embedded in paraffin for storage. Clinicopathological data were also collected. In addition, we extracted proteins from 48 pairs of fresh gastric cancer tissues and stored them at − 80 °C for western blotting. All patients signed informed consents, and all procedures were in accordance with the Standards of Ethics Committee of China Medical University.

### Immunohistochemistry

Immunohistochemistry was carried out according to previously published methods [14, 15]. The primary antibody against LMOD1 was purchased from Accepta (China). The percent positive cells and staining intensity were used to determine the degree of expression. The percentage of positive cells was divided into five groups: 0: < 5%; 1:5 – 25%; 2:25 – 50%; 3:50 – 75%; 4: > 75%. The staining intensity was divided into four groups: 0: colorless; 1: light yellow; 2: brown; 3: dark brown. Scores 0-4 were considered low expression, and scores 6-12 were considered high expression.

### In vivo gastric cancer peritoneal dissemination assay

Gastric cancer peritoneal dissemination experiments were carried out according to previous experimental methods [15]. 10 Blab / C nude mice were randomly divided into two groups with five mice in each group. A mixed suspension of 2× 10^6^ SGC-7901 shCtrl/ shLMOD1 cells and 5 × 10^5^ HPMCs and SGC-7901 and HPMCs were injected intraperitoneally. Mice were euthanized 6 weeks after tumor inoculation. The location and number of abdominal macroscopic lesions were recorded, and the tumors were weighed. All methods were in accordance with the requirements of the Animal Committee of China Medical University.

### Statistical analysis

SPSS 20.0 and GraphPad Prism 7.0 were used for data statistics and analysis. A Student’s t-test was used to compare the differences between the two means, and the correlation with clinicopathological factors was analyzed using a chi-square test. The Kaplan – Meier method was used to calculate the difference in overall survival, and X-tile was used to identify Optimal cut-off value. *P* < 0.05 was considered statistically significant.

## Results

### Construction and analysis of WGCNA from GEO databases

**Fig. 1:**
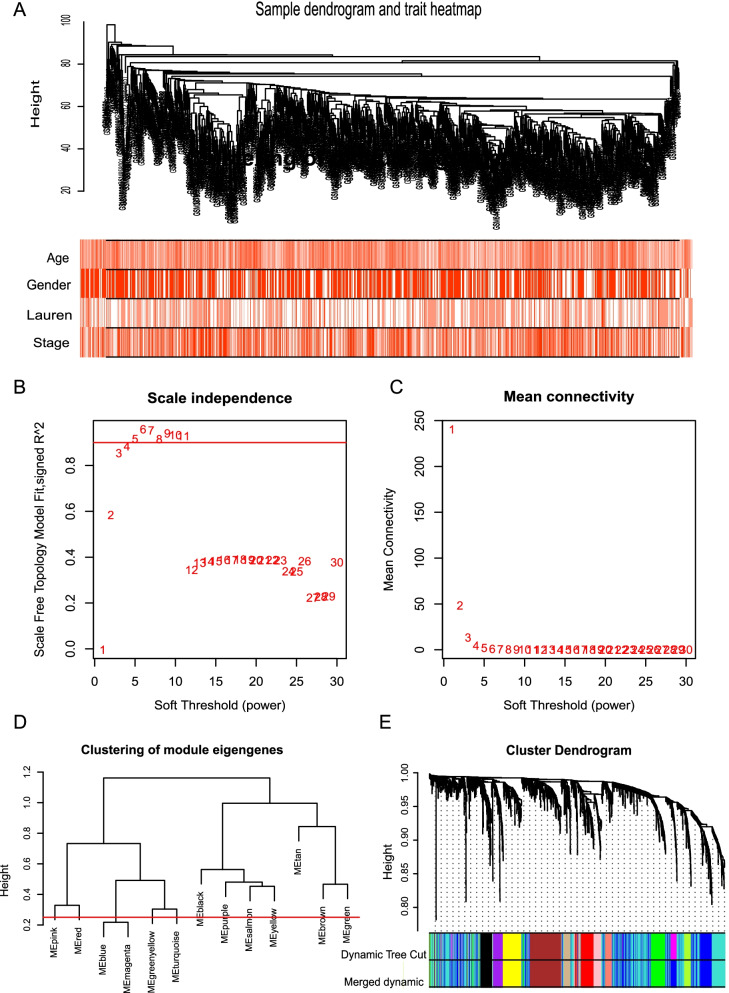
Data processing and construction of WGCNA. **A** Clustering dendrogram of the clinical traits. **B**, **C** Calculation of the soft threshold by scale independence and mean connectivity. When the soft threshold was 3, the scale free topology fitting index R2 was 0.853. **D** Clustering dendrogram of genes based on topological overlap

**Fig. 2:**
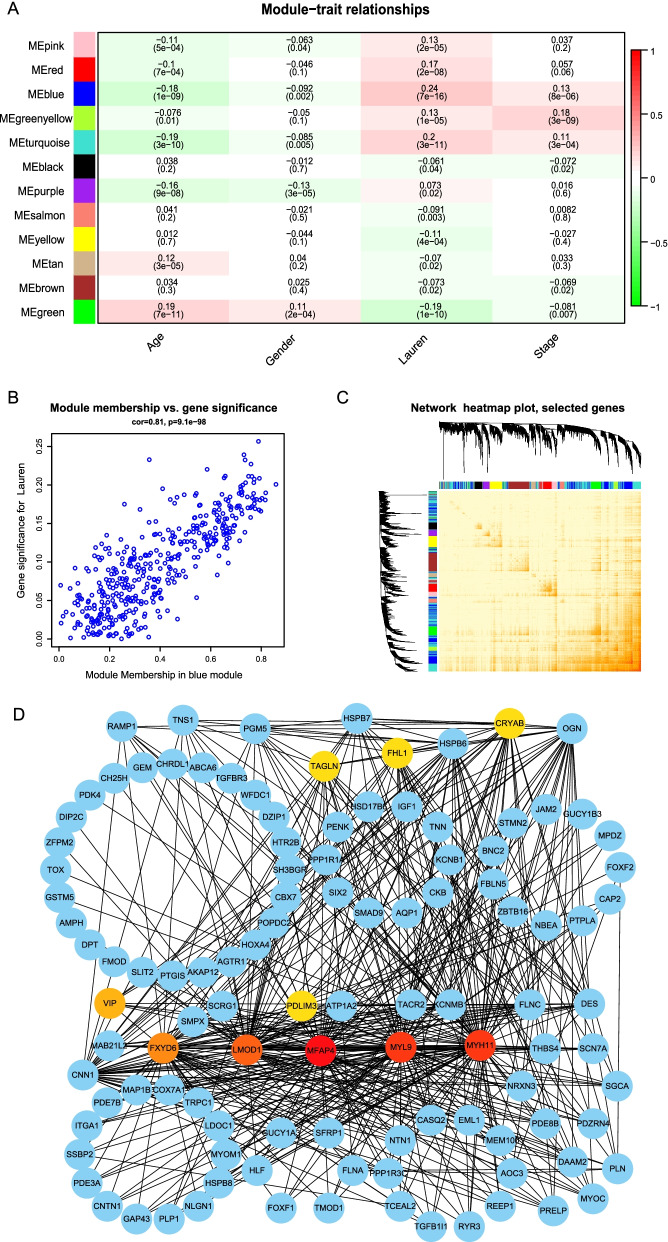
Analysis of the WGCNA module. **A** Heatmap of modules for gene-traits relationship. Red represents a positive correlation, and green represents a negative correlation. **B** The correlation between blue module membership and Lauren classification was significant. The correlation coefficient was 0.81. **C** Network heatmap plot. **D** Utilization of Cytoscape to visualize the relationship between genes in the blue module. Six key genes were selected from the network using the Maximal Clique Centrality algorithm

We extracted gene expression information from 8135 genes from five gastric cancer GEO datasets, analyzed the expression of each gene in 1001 patient samples using ANOVA, and took the top 25% of genes (2035) for subsequent WGCNA analysis. The expression and clinical information from 1001 patients were analyzed using cluster analysis (Fig. 1A), followed by calculation of the soft threshold. When the soft threshold was set at three, the scale-free topology fitting index (R2) was 0.853 (Fig. 1B and C). Finally, 12 co-expression modules were obtained (Fig. 1D and E). The correlation coefficient between the blue module and the Lauren classification was 0.24 (Fig. 2A). The genes in the blue module were selected for analysis, and the module membership and the significance of genes were calculated. The correlation between the two was 0.81, and the *P* value was statistically significant (Fig. 2B and C). The relationship between genes in the blue module was visualized using Cytoscape, and the relationship between genes with weighted values in the top 500 were selected for input into Cytoscape. The Maximal Clique Centrality (MCC) algorithm in the plug-in was used to calculate. The higher the weight of the network in the module, the greater the representative effect, and six key genes (MFAP4, MYL9, MYH11, LMOD1, FXYD6 and VIP) were selected from the network (Fig. 2D).

### LMOD1 is identified as an oncogene and is associated with Lauren classification

**Fig. 3:**
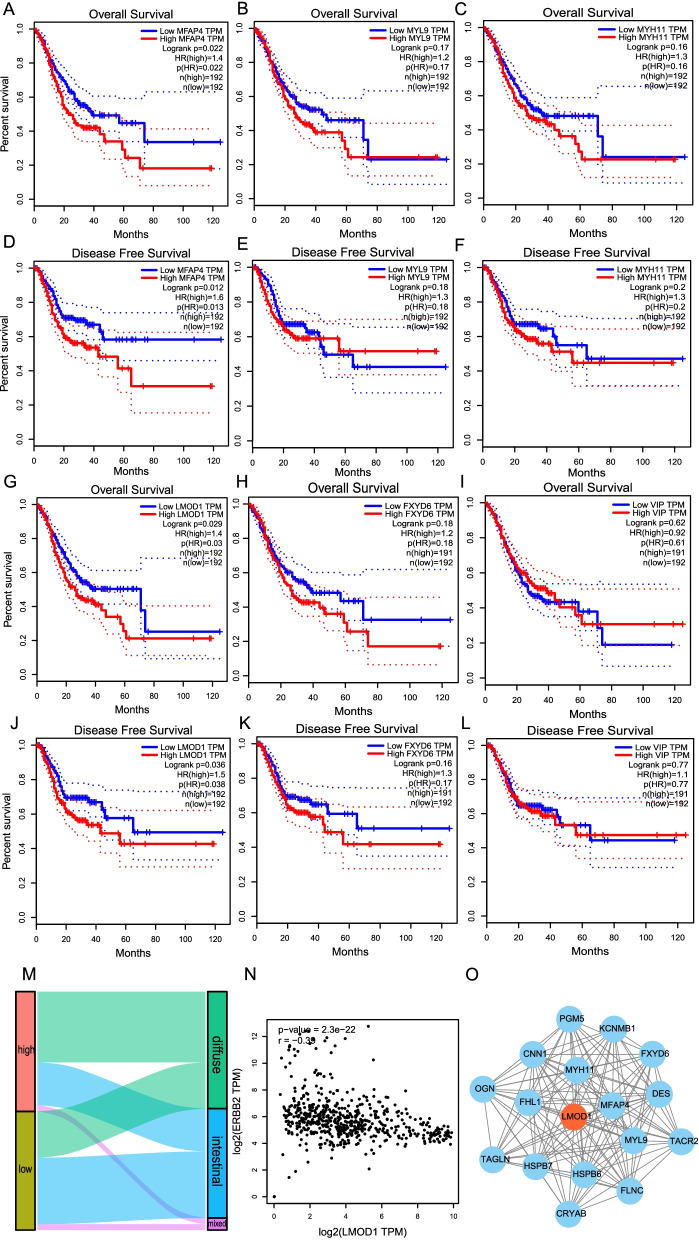
Identification of the key gene related to Lauren classification. **A**-**L** Overall survival and disease-free survival curves for six key genes in the TCGA and GSE62254 databases (MFAP4, MYL9, MYH11, LMOD1, FXYD6, VIP). **M** Relationship between gene expression of LMOD1 and Lauren classification of patients. **N** The correlation between LMOD1 and HER-2 (ERBB2) expression in TCGA and GEO databases. **M** Analysis with the MCOD algorithm yielding related sub-modules of LMOD1 in blue cyan

To identify the genes with a high correlation between expression and Lauren classification, we used the TCGA database to measure the OS and PFS of hub genes in the blue module. Patients with high MFAP4 and LMOD1 expression had a worse prognosis, while the other four genes were not statistically correlated with prognosis (Fig. 3A-L). It has previously been reported that MFAP4 is a cancer-promoting gene with a positive effect on the development of gastric cancer. Since, LMOD1 had not been previously studied in relation to gastric cancer, this gene was chosen for further study. By analyzing the relationship between LMOD1 expression and Lauren classification in GEO databases, we found that patients with high LMOD1 expression had diffuse gastric cancer, while patients with low LMOD1 expression were more likely to have intestinal gastric cancer (Fig. 3M). The expression of LMOD1 was negatively correlated with HER-2 in GEO and TCGA databases (Fig. 3N). We chose the genes in the blue module that were highly related to LMOD1 to construct a PPI network (Fig. 3O).

### LMOD1 regulates the migration of gastric cancer cells through the FAK-Akt/mTOR pathway in vitro

**Fig. 4:**
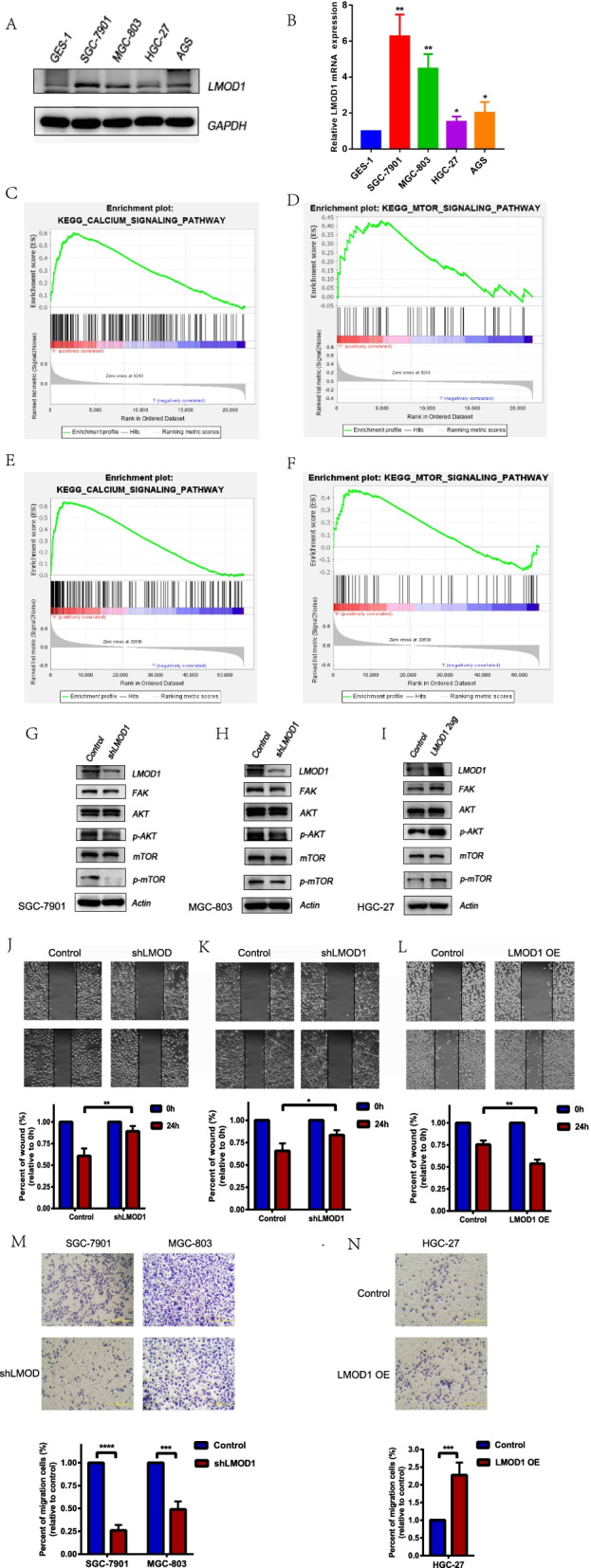
LMOD1 enhanced the migration ability of gastric cancer cells through the FAK-Akt/mTOR pathway. **A** The expression of LMOD1 in cell lines was measured using western blots. **B** The expression of LMOD1 in cell lines was measured by RT-qPCR. **C**-**F** A GSEA analysis of LMOD1 enriched pathways in TCGA and GSE62254 databases. **G**, **H** Western blotting was used to measure protein markers of the FAK-Akt/mTOR pathway in Control or LMOD1- silenced SGC-7901 and MGC-803 cell lines. **I** Western blotting was used to measure protein markers of the FAK-Akt/mTOR pathway in the Control or LMOD1-overexpressed HGC-27 cell line. **J**, **K** The migration ability of cells was detected using a scratch test. **M**, **N** The migration ability of cells was measured using a transwell test. The membrane was cut out due to the different secondary antibodies incubated. *, *P* < 0.05, **, *P* < 0.01, ***, *P* < 0.001, ****, *P* < 0.0001

The expression of LMOD1 protein and mRNA was detected in cell lines. The expression of LMOD1 in GES-1 cells was lower than that in gastric cancer cell lines, further supporting that LMOD1 is a cancer-promoting gene (Fig. 4A and B). Moreover, LMOD1 was highly expressed in SGC-7901 and MGC-803 cells, and lowly expressed in HGC-27 and AGS cells. To explore the role of LMOD1 in the occurrence and development of gastric cancer, we used TCGA and GSE62254 databases to analyze LMOD1 with GSEA. LMOD1 was enriched in Ca + signaling pathways and the mTOR signaling pathway (Fig. 4C-F) in both databases. We next generated LMOD1-silenced (shLMOD1) SGC-7901 and MGC-803 cell lines and LMOD1-overexpressing (LMOD1-OE) HGC-27 cell lines. The expression of FAK, p-Akt and p-mTOR in shLMOD1 cells was decreased (Fig. 4G and H), while the expression of FAK, p-Akt and p-mTOR in LMOD1 OE cells increased (Fig. 4I), indicating that LMOD1 can further affect the phosphorylation of Akt and mTOR by altering the expression of FAK. Meanwhile, compared with the control group, the migration ability of shLMOD1 cells was significantly decreased (Fig. 4J, K and M), while the migration ability of LMOD1 OE cells was significantly increased (Fig. 4L and N).

### LMOD1 promotes peritoneal metastasis of gastric cancer cells by regulating EMT

**Fig. 5:**
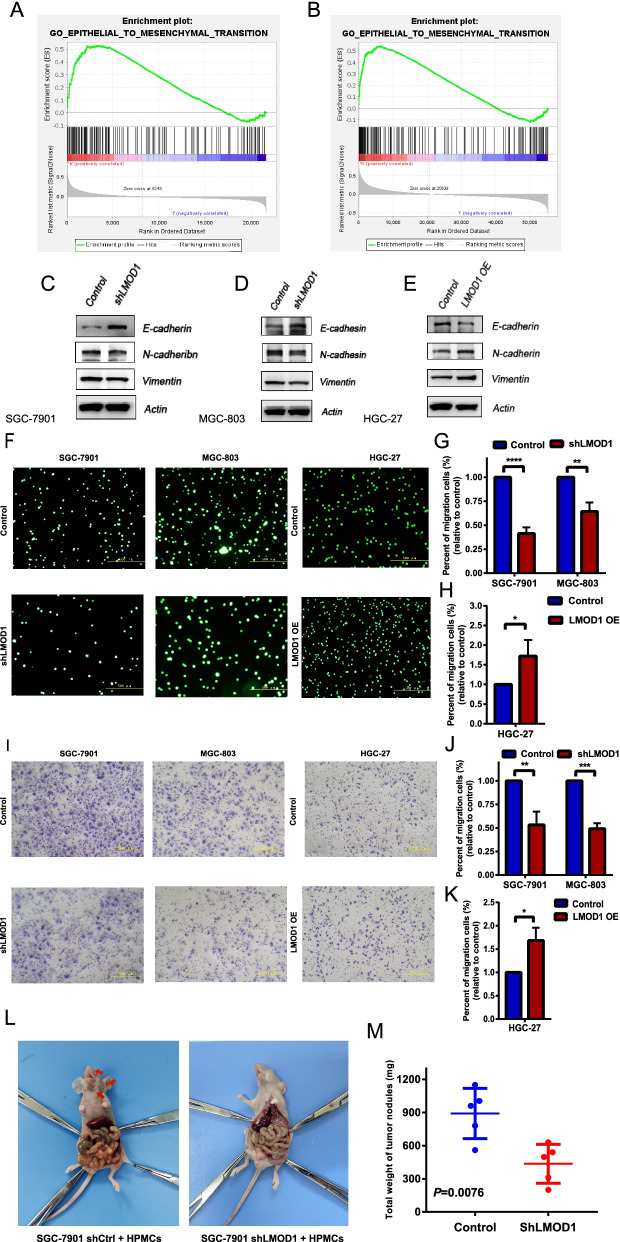
LMOD1 promoted peritoneal metastasis of gastric cancer cells by regulating the epithelial-mesenchymal transition. **A**, **B** GSEA analysis of LMOD1 enriched pathways in TCGA and GSE62254 databases. **C**, **D** Western blotting was used to verify the regulation of epithelial-mesenchymal transition (EMT)-related proteins in Control or shLMOD1 SGC-7901 and MGC-803 cell lines. **E** Western blotting was used to verify the regulation of EMT-related proteins in Control/\ or overexpressed-LMOD1 HGC-27 cell lines. **F**-**H** The adhesion ability of cells was verified using an adhesion test. **I**-**K** An invasion assay was used to verify the ability of peritoneal infiltration. **L**, **M** The effect of LMOD1 silencing on invasion and migration of gastric cancer cells in SGC-7901 cells was verified by intraperitoneal metastasis model in nude mice. We cut the membrane to facilitate the better hybridization of antibodies and avoid the interaction of antibodies. *, *P* < 0.05, **, *P* < 0.01, ***, *P* < 0.001, ****, *P* < 0.0001

Patients with diffuse gastric cancer often suffer from peritoneal metastasis, which greatly affects the prognosis of gastric cancer patients. Studies have shown that mTOR phosphorylation can affect downstream EMT and further promote the invasion and metastasis of gastric cancer cells [16, 17]. To answer whether LMOD1 could promote peritoneal metastasis of gastric cancer by affecting EMT, we predicted that LMOD1 could be enriched in the EMT pathway in the TCGA and GSE62254 databases (Fig. 5A and B). We then measured the expression of EMT-related protein markers in LMOD1-silenced and LMOD1-OE cells. The results showed that compared with the control group, the levels of E-cadherin in LMOD1-silenced cells were increased, and the levels of N-cadherin and vimentin were decreased (Fig. 5C-E). In contrast, the levels of E-cadherin in LMOD1-OE cells were decreased, and the levels of N-cadherin and vimentin were increased. Furthermore, in adhesion and invasion tests, compared with the control group, the adhesion and penetration of LMOD1-silenced cells were decreased, while the adhesion and penetration of LMOD1-OE cells were increased (Fig. 5F-K). In the model of peritoneal metastasis in nude mice, the number and weight of tumor nodules from SGC-7901 shLMOD1 cells were significantly lower than that of the control group (Fig. 5L, M; Supplement figure 1).

### Expression and survival analyses of LMOD1 in clinical tissue samples

**Fig. 6:**
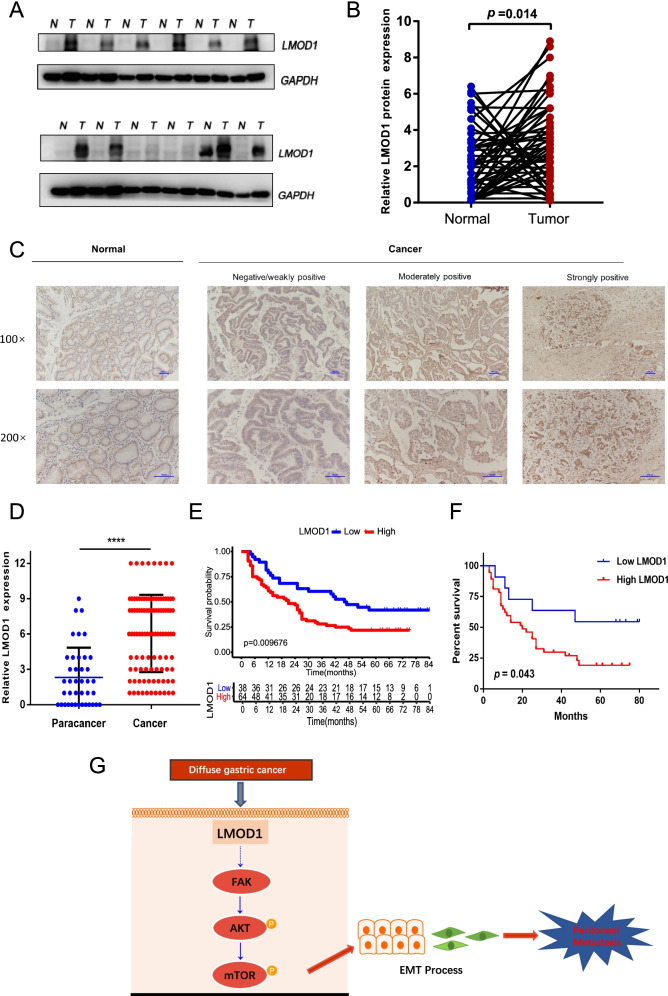
Expression and survival significance of LMOD1 in clinical tissue samples. **A**, **B** Western blotting was used to measure the protein expression of LMOD1 in 48 pairs of gastric cancer tissues. **C**, **D** LMOD1 protein expression was measured by immunohistochemistry in 102 cases of gastric cancer tissues and 40 cases of adjacent tissues. **E** Overall survival curve of LMOD1in gastric cancer. **F** Overall survival curve of LMOD1 in diffuse gastric cancer. **G** Schematic diagram showed that the regulatory effect of LMOD1 on peritoneal metastasis in diffused gastric cancer. The membrane was cut out due to the different secondary antibodies incubated. ****, *P* < 0.0001

To explore the expression and prognostic significance of LMOD1 in clinical tissues of patients with gastric cancer, we measured the protein expression of LMOD1 in 48 pairs of gastric cancer and adjacent tissue samples. The expression of LMOD1 in cancer tissues was significantly higher than that in adjacent tissues, and the difference was statistically significant (*P* = 0.014; Fig. 6A and B). Next, we examined the expression of LMOD1 in 102 gastric cancer tissues and 40 paracancerous tissues using immunohistochemistry (Fig. 6C), which also showed that the expression of LMOD1 in gastric cancer tissues was higher than that in paracancerous tissues (Fig. 6D). According to the LMOD1 immunohistochemical scores, 102 patients with gastric cancer were divided into a low-expression group (38/102) or a high-expression group (64/102). Survival analysis showed that patients with high expression of LMOD1 had worse prognosis than patients with low LMOD1 expression (Fig. 6E), Meanwhile, in diffuse gastric cancer, the survival time of patients with high expression of LOMD1 was significantly shorter than that of patients with low expression (Fig. 6F). The clinicopathological analysis showed that the expression of LMOD1 was positively correlated with Borrmann type, Lauren type and T stage (Table 1). The high expression of LMOD1 accounted for 59.4% in patients with diffused gastric cancer and 28.9% in patients with intestinal type.

**Table 1:** Correlation between LMOD1 expression and clinicopathologic characteristics in GC patients

	LMOD1 expression		
Factor	Low (***n*** = 38)	High (***n*** = 64)	***P*** value
Sex			0.361
Male	26	38	
Female	12	26	
Age			0.161
< 60	25	33	
≥ 60	13	31	
Tumor location			0.271
Upper	3	7	
Middle	5	16	
Lower	30	41	
Tumor size			0.794
< 4 cm	11	17	
≥ 4 cm	27	47	
Borrmann type			**0.020**
I-III	22	22	
IV	16	42	
Histological type			
Well/moderate	16	29	0.752
Poor	22	35	
Lauren type			**0.003**
intestinal	27	26	
diffused	11	38	
T stage			**0.008**
T2-3	24	20	
T4	14	44	
N stage			0.682
N0-2	20	31	
N3	18	33	

## Discussion

The Lauren classification designates special types of gastric cancer. Compared with the diffuse type, the intestinal type is less prone to peritoneal metastasis and has a better prognosis. Therefore, it is especially important to identify the key genes that determine Lauren classification typing for targeted therapy of gastric cancer. In this paper, we constructed a WGCNA model by analyzing the gene expression and clinical information of patients in GEO gastric cancer databases, and analyzed the blue cyan modules related to Lauren classification. Eventually, LMOD1 was selected as the target of our analysis. In vitro and in vivo experiments verified that LMOD1 could affect the migration ability of gastric cancer cells by regulating the FAK-Akt/mTOR pathway, which can alter the EMT of cells to promote the occurrence of peritoneal metastasis in gastric cancer (Fig. 6G).

Peritoneal metastasis is an independent prognostic factor in gastric cancer [18, 19]. About 20% of patients are diagnosed with peritoneal metastasis preoperatively or intraoperatively, and the median survival time is only 6-7.5 months [20]. Diffuse gastric cancer is most likely to develop peritoneal metastasis, accounting for about 37%, while intestinal gastric cancer most likely to develop distant metastasis [21]. The adhesion and invasion ability of gastric cancer cells can be increased through EMT, which permeates the barrier of mesothelial cells, allowing for entry into the abdominal cavity to produce peritoneal metastasis. However, due to the limitations of technology and surgical methods, researchers can rarely obtain tissue samples from patients with peritoneal metastasis of gastric cancer. In this study, we extracted and analyzed gene modules highly related to Lauren classification by using clinicopathological factors and gene expression in the GEO database and WGCNA algorithm, to further predict and explore the mechanism of peritoneal metastasis of gastric cancer.

Constructing co-expression modules using the WGCNA algorithm has been widely used to identify potential key or prognostic genes. In a study by Wan et al. data from melanoma patients from the TCGA was analyzed using WGCNA, yielding 21 modules, and four key genes related to melanoma recurrence [22]. Zhai et al. used data from the E-GEOD-39582 colorectal cancer microarray to obtain 434 differentially genes. After performing a WGCNA analysis, they obtained 5 differential genes related to recurrence and prognosis [23]. In the current study, we used the WGCNA algorithm to find gene enrichment modules associated with diffuse and intestinal gastric cancer and selected the hub gene, LMOD1, to determine the molecular mechanism of Lauren classification. The research and development of the WGCNA method was originally applied to the development of space technology. However, WGCNA can now play an important role in exploring the specific mechanism of tumor occurrence and development through interdisciplinary research.

The LMOD1 protein has a putative transmembrane domain and two kinds of repeated blockades. It was initially identified as a 64 kDa autoantigen in a serum screening study of Hashimoto’s thyroiditis patients. It is reported that LMOD1 is a new target gene of smooth muscle cell-restricted serum response factor/myocardial protein [12]. In the cancer setting, Zhao et al. demonstrated that LMOD1 could be used as a key gene to determine the diagnosis and prognosis of early-onset colorectal cancer [24]. Kawahara et al. showed that the expression of LMOD1 was significantly different between low-grade and high-grade of prostate cancer, which could be used as a biological marker for the diagnosis of prostate cancer [25]. In this study, a GSEA analysis and western blotting demonstrated that LMOD1 could alter the migration ability of gastric cancer cells by regulating FAK-Akt / mTOR pathway. In vivo and in vitro experiments further confirmed that LMOD1 could promote peritoneal metastasis by regulating EMT of gastric cancer cells.

The Lauren classification is one of the most important phenotypes of gastric cancer. HER-2 as a therapeutic target for breast cancer has been widely confirmed [26, 27]. In gastric cancer patients, the HER-2 positive rate is about 10 - 30%. ToGA study showed that for HER-2-positive gastric cancer patients, trastuzumab combined with chemotherapy resulted in the longest overall survival and enhanced efficacy for intestinal gastric cancer, with the worst prognosis being for diffuse gastric cancer patients. Meanwhile, the positive rate of HER-2 in intestinal patients was significantly higher than that of diffuse type patients [4]. Qiu et al. reported that the prognosis of patients with HER-2-negative intestinal gastric cancer was better than that of patients with HER-2-positive diffuse gastric cancer [28]. Our results showed that LMOD1 was highly expressed in diffuse gastric cancer and negatively correlated with HER-2 expression. However, the relationship between HER-2 and prognosis in gastric cancer is not clear. Based on these studies, it is therefore critical to explore the biological functional genes that determine development of the intestinal type and diffuse type of gastric cancer, to provide a promising target for the future treatment of gastric cancer.

“Seed and soil theory” is a highly recognized theoretical mechanism of peritoneal metastasis of gastric cancer [29, 30]. The abscission of cancer cells and the change of extracellular matrix components promotes the implantation of cancer cells in the peritoneum. EMT is the main mechanism for the peritoneal metastasis of cancer cells. It is reported that the exosomes in malignant ascites can promote the EMT of gastric cancer cells and increase the diffusion degree of gastric cancer cells in the abdominal cavity [31]. Chen et al. showed that FNDC1 can be closely related to the peritoneal metastasis of gastric cancer by regulating the EMT pathway of cells [32]. Moreover, studies have shown that FAK activation is an important upstream protein regulating EMT [33, 34]. Our results suggest that LMOD1 can upregulate the expression of FAK, activate the phosphorylation level of the Akt / mTOR pathway, promote EMT in gastric cancer cells, increase cell invasion and migration, and promote the occurrence and development of peritoneal metastasis of gastric cancer.

To summarize, we used clinical patient information and gene expression data from GEO databases of gastric cancer to build a WGCNA model. We then selected the modules relating to Lauren classification, and identified the oncogene LMOD1 in the module for further study. LMOD1 was found to regulate the FAK-Akt/mTOR pathway to induce EMT of gastric cancer cells, to further increase invasion and adhesion of gastric cancer cells and promote peritoneal metastasis of gastric cancer cells. These results indicate that LMOD1 could sever as a novel biomarker and therapeutic target for gastric cancer.

## Supplementary Information

**Additional file 1.**

## Data Availability

The data that support the findings of this study are openly available in GEO databases and The Cancer Genome Atlas database (https://www.ncbi.nlm.nih.gov/gds, https://www.cancer.gov/aboutnci/organization/ccg/research/structural-genomics/tcga).
