# Supplementary material for: LMOD1, an oncogene associated with Lauren classification, regulates the metastasis of gastric cancer cells through the FAK-AKT/mTOR pathway
Source: BMC Cancer. 2022 Apr 30;22:474. doi: 10.1186/s12885-022-09541-0 (PMC9055720; doi:10.1186/s12885-022-09541-0)

The nucleotide sequence for the LMOD1 overexpression plasmid :

ATGTCTAGAGTAGCCAAATATCGCCGGCAGGTGAGTGAAGACCCCGACATCGACAGCCTGCTG
GAGACCCTGTCTCCCGAGGAGATGGAGGAGCTGGAGAAGGAGCTGGACGTGGTGGACCCAGA
CGGGAGTGTTCCCGTGGGGCTGCGGCAGAGAAACCAGACGGAGAAACAGTCCACGGGTGTGT
ACAACCGGGAGGCCATGCTCAACTTCTGTGAAAAGGAGACCAAGAAACTTATGCAGAGGGAGA
TGTCCATGGATGAAAGCAAGCAAGTGGAGACCAAGACAGATGCCAAGAATGGAGAGGAAAGG
GGCAGAGATGCCAGCAAAAAAGCCCTGGGCCCCAGACGGGACTCAGATCTGGGGAAGGAGCC
AAAGAGGGGTGGTTTAAAGAAAAGCTTCTCTAGAGACAGAGATGAAGCTGGTGGCAAGAGTGG
CGAGAAGCCCAAGGAGGAGAAGATCATCCGGGGCATTGACAAGGGCCGGGTCAGGGCTGCAG
TGGATAAGAAGGAGGCAGGGAAGGATGGGAGAGGAGAGGAGAGGGCAGTGGCCACCAAGAA
GGAAGAGGAGAAGAAAGGGAGTGACAGGAACACAGGCTTGAGCAGGGACAAGGATAAAAAG
AGAGAGGAGATGAAGGAGGTGGCCAAGAAAGAGGATGATGAGAAGGTAAAAGGGGAGCGTA
GGAACACAGACACCAGAAAAGAGGGTGAGAAGATGAAAAGAGCAGGTGGGAACACAGACAT
GAAAAAGGAGGATGAGAAGGTAAAAAGAGGAACTGGGAACACAGACACCAAAAAGGACGAT
GAAAAAGTCAAGAAGAATGAACCCTTACATGAAAAGGAAGCCAAGGATGACAGCAAGACCAA
AACACCCGAGAAACAGACGCCCAGTGGCCCCACCAAGCCCTCTGAAGGACCGGCCAAGGTGG
AGGAGGAGGCAGCTCCCAGCATATTTGATGAGCCTCTGGAGAGAGTGAAGAACAATGACCCCG
AGATGACTGAGGTGAACGTCAACAACTCAGACTGCATCACAAATGAGATCTTGGTCCGGTTTAC
TGAGGCTCTGGAGTTCAACACTGTGGTTAAGCTGTTCGCCTTGGCCAACACGCGAGCCGATGAC
CACGTGGCCTTTGCCATTGCCATCATGCTCAAGGCCAACAAGACCATCACCAGCCTCAACCTGG
ACTCCAACCACATCACAGGCAAAGGCATCCTGGCCATCTTCCGGGCCCTCCTCCAGAACAACA
CGCTGACCGAGCTCCGCTTCCACAACCAGCGACACATCTGTGGAGGCAAGACGGAGATGGAG
ATCGCCAAGCTGCTGAAGGAGAATACTACCCTGCTCAAGCTGGGCTACCATTTTGAGCTGGCCG
GGCCCCGAATGACTGTCACCAATCTGCTCAGCCGCAACATGGACAAGCAGAGACAAAAGCGG
CTGCAGGAGCAAAGGCAGGCACAGGAAGCCAAGGGAGAGAAGAAGGATCTGCTGGAGGTAC
CCAAGGCCGGGGCCGTGGCTAAGGGCTCCCCAAAACCTTCACCTCAACCATCTCCAAAGCCCT
CTCCAAAGAACTCACCCAAAAAAGGGGGTGCTCCAGCTGCCCCACCACCCCCTCCCCCTCCCTT
GGCTCCACCCCTTATCATGGAGAACCTGAAGAATTCACTCTCACCAGCTACCCAGAGGAAGATG
GGAGACAAAGTCCTCCCTGCCCAGGAGAAGAACTCCCGTGACCAGCTATTGGCTGCCATCCGC
TCCAGCAACCTCAAGCAGCTCAAGAAGGTGGAAGTGCCCAAACTGCTTCAGTAG

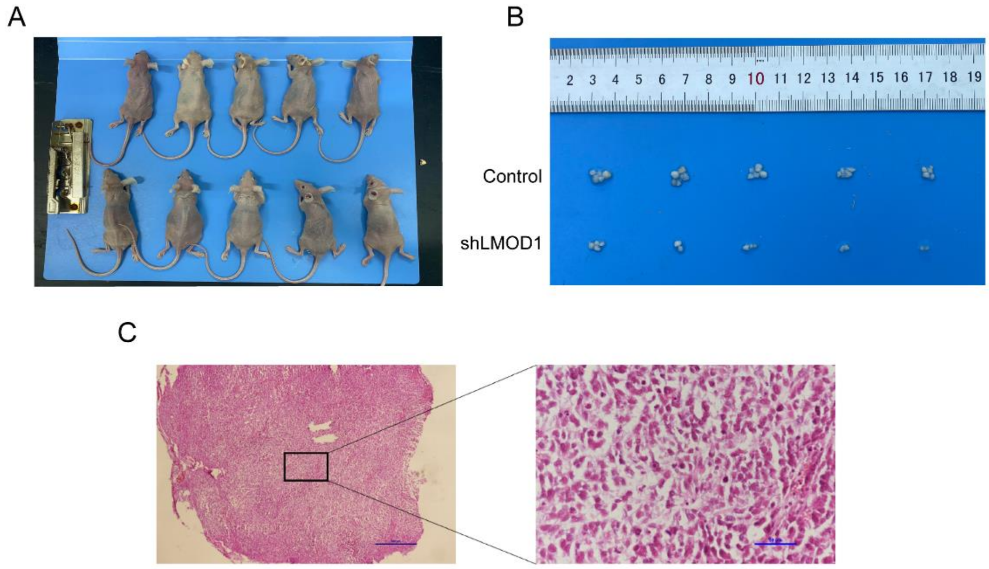

Supplement figure 1. Peritoneal metastasis model in nude mice.

A. General picture of nude mice. B. Solid tumors in abdominal cavity of nude mice. C. He staining of solid tumors.

Supplementary Figure_Original Western blots

Figure 4A

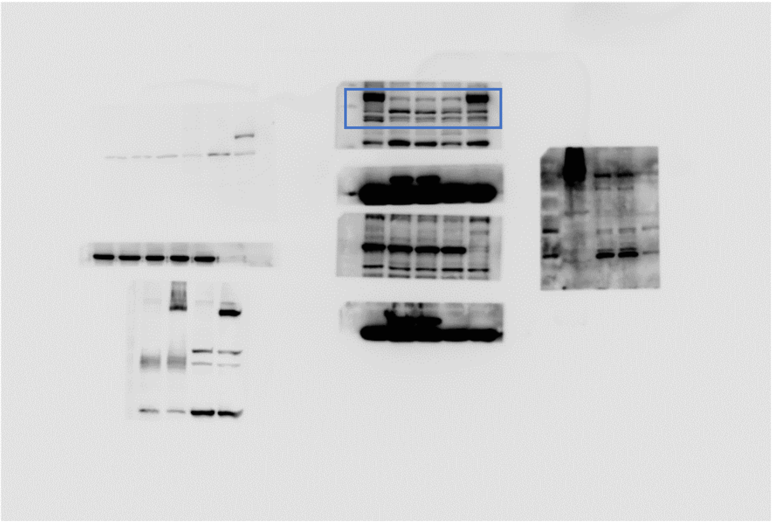

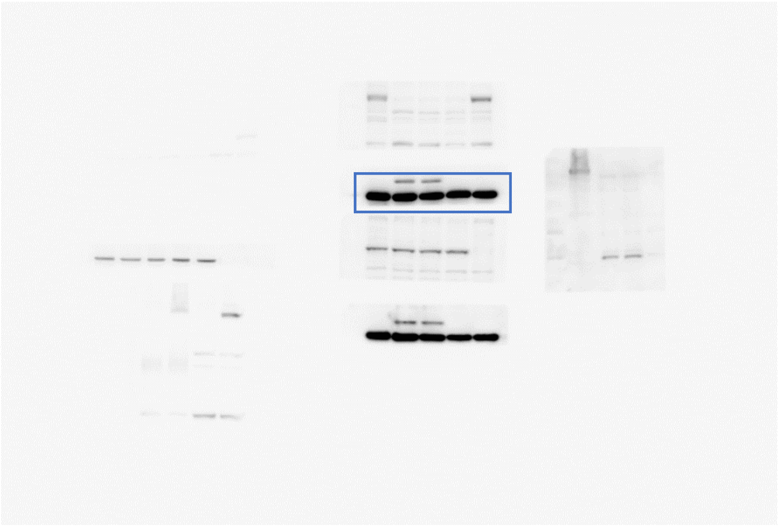

Figure 4G

LMOD1

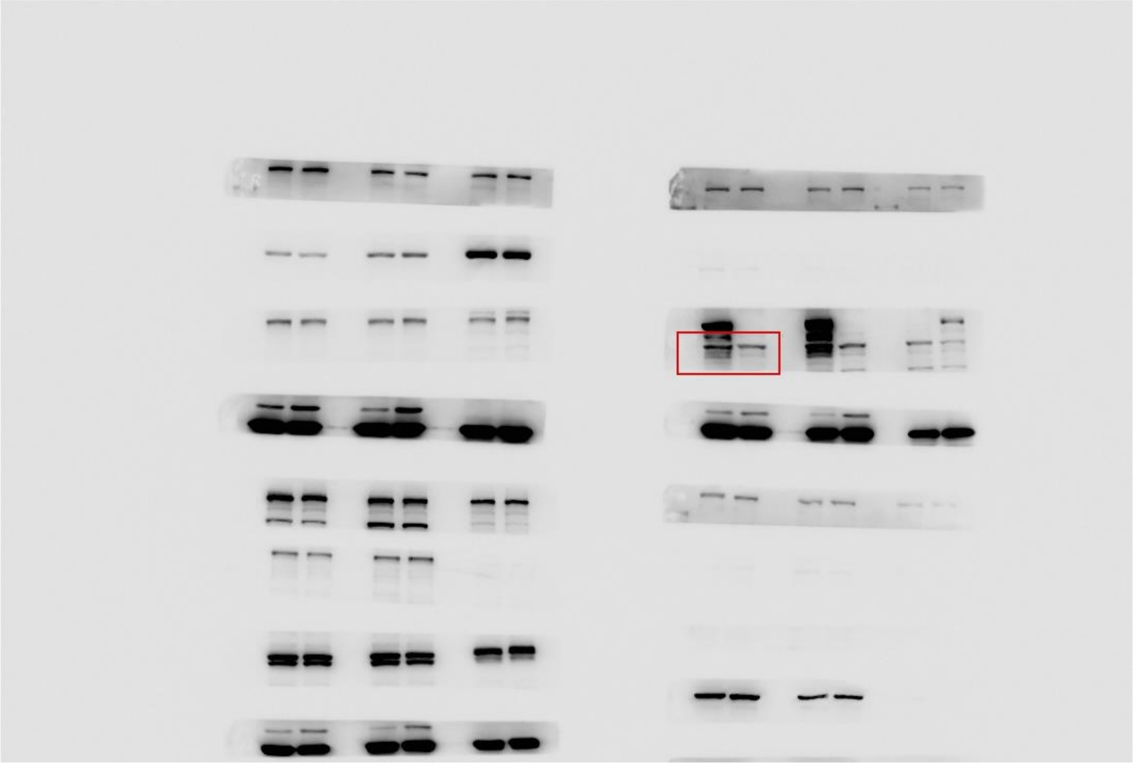

FAK

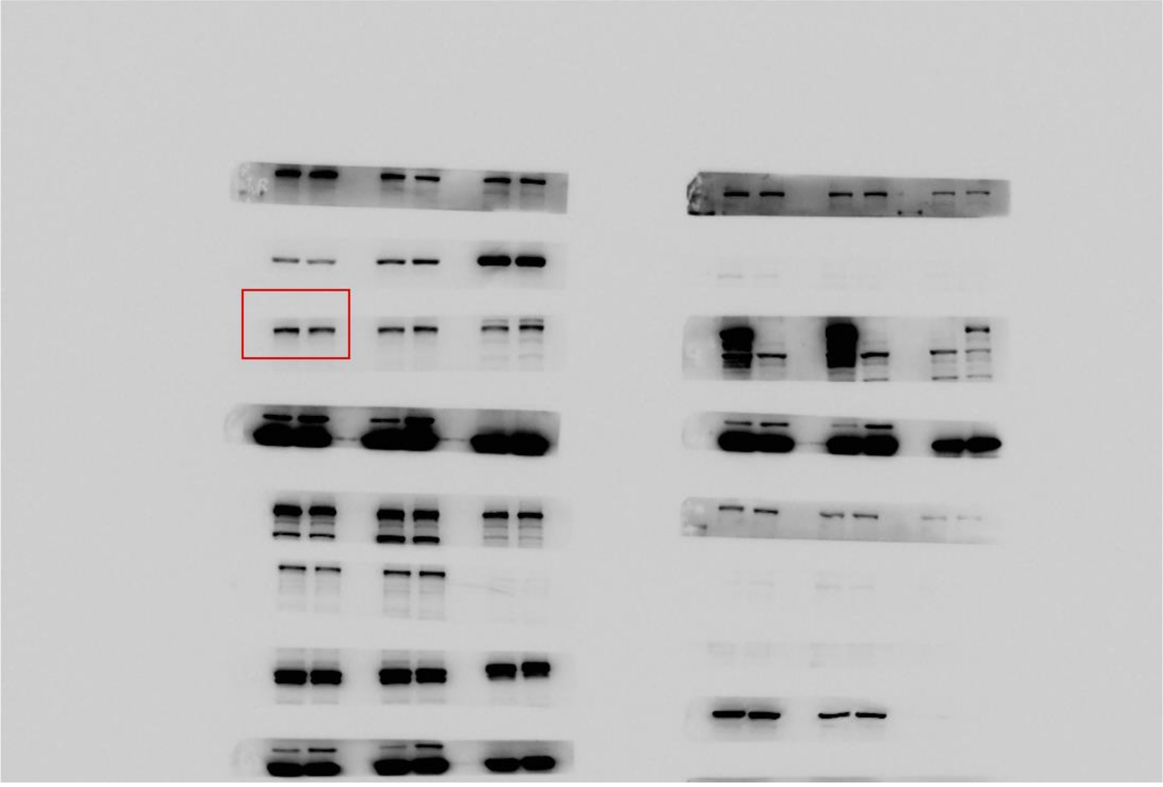

AKT

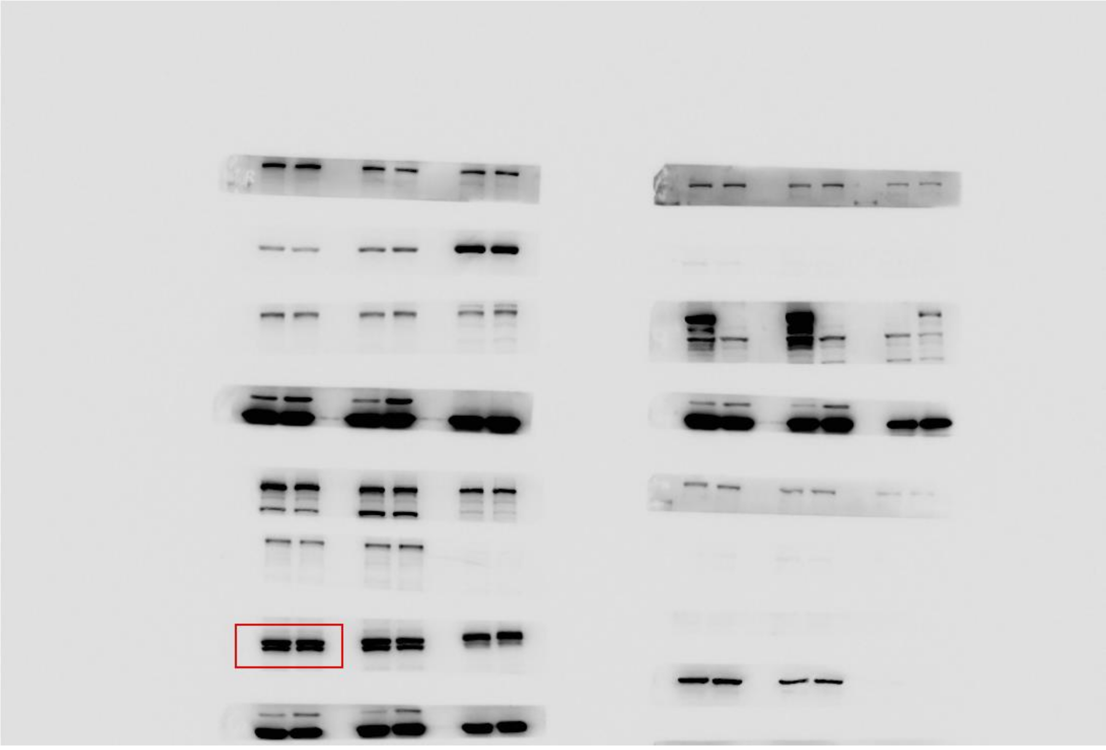

p-AKT

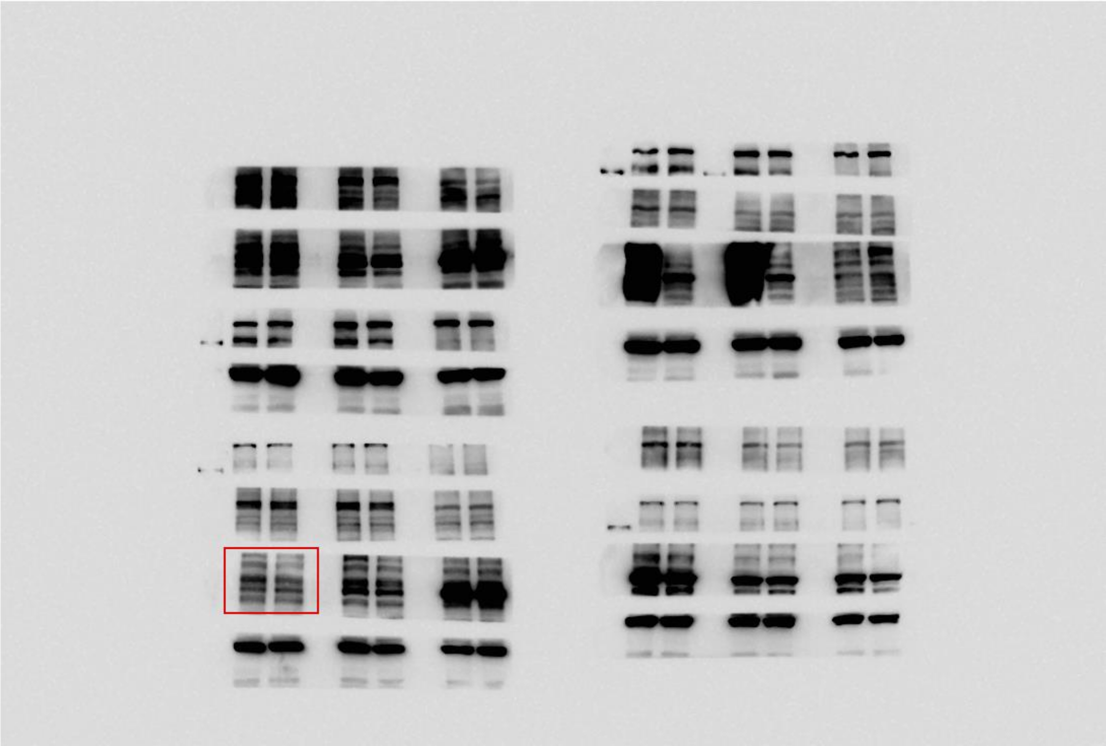

mTOR

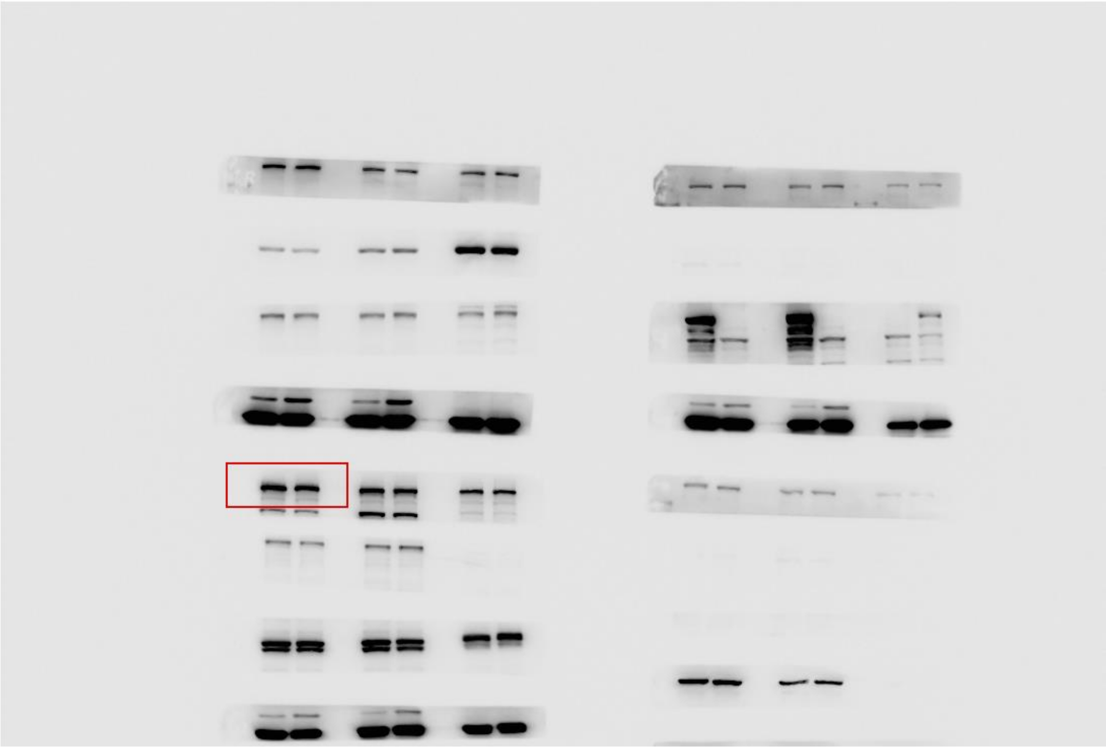

p-mTOR

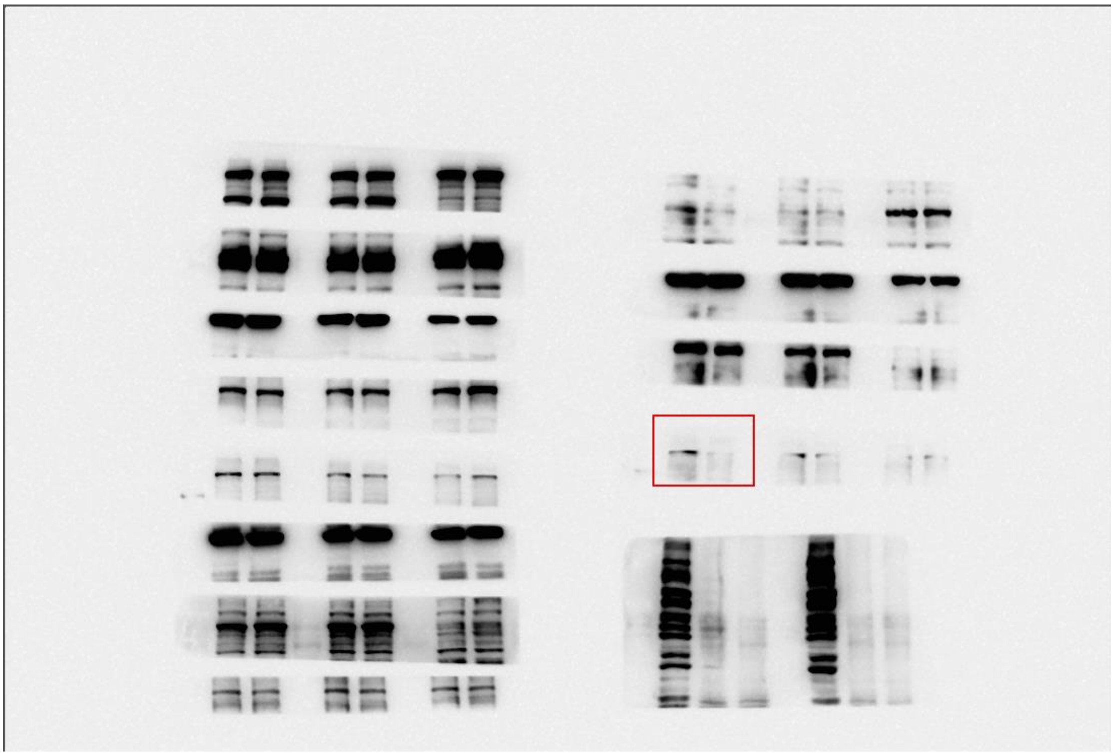

Actin

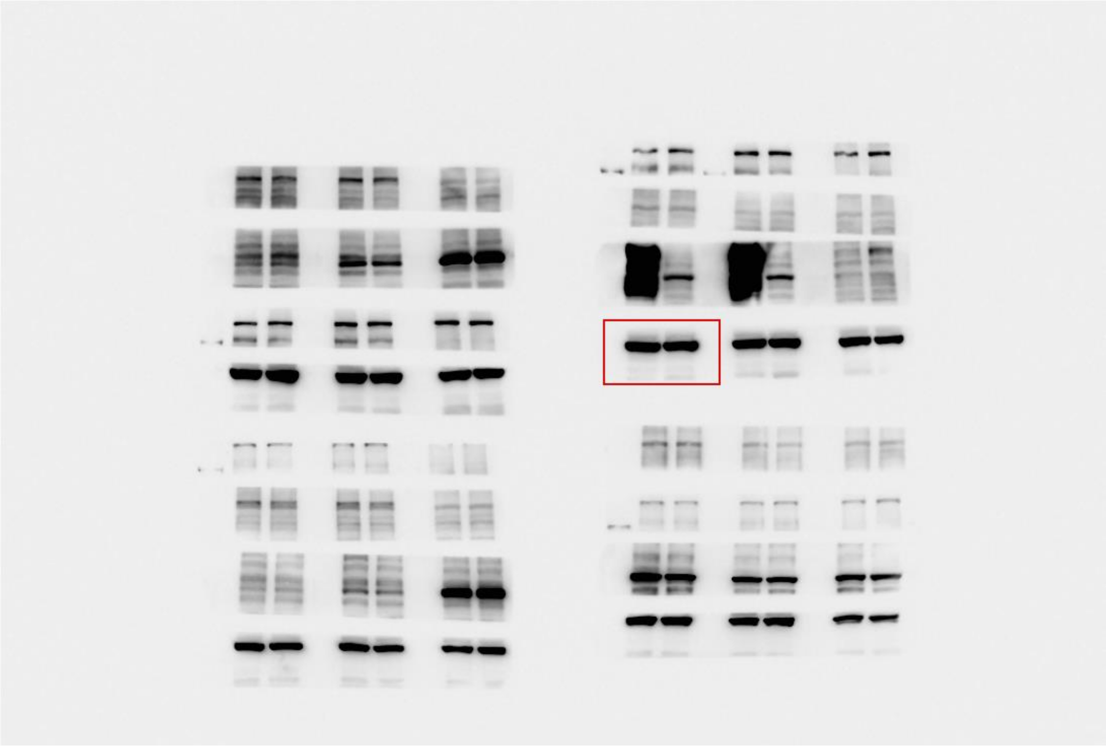

Figure 4H

LMOD1

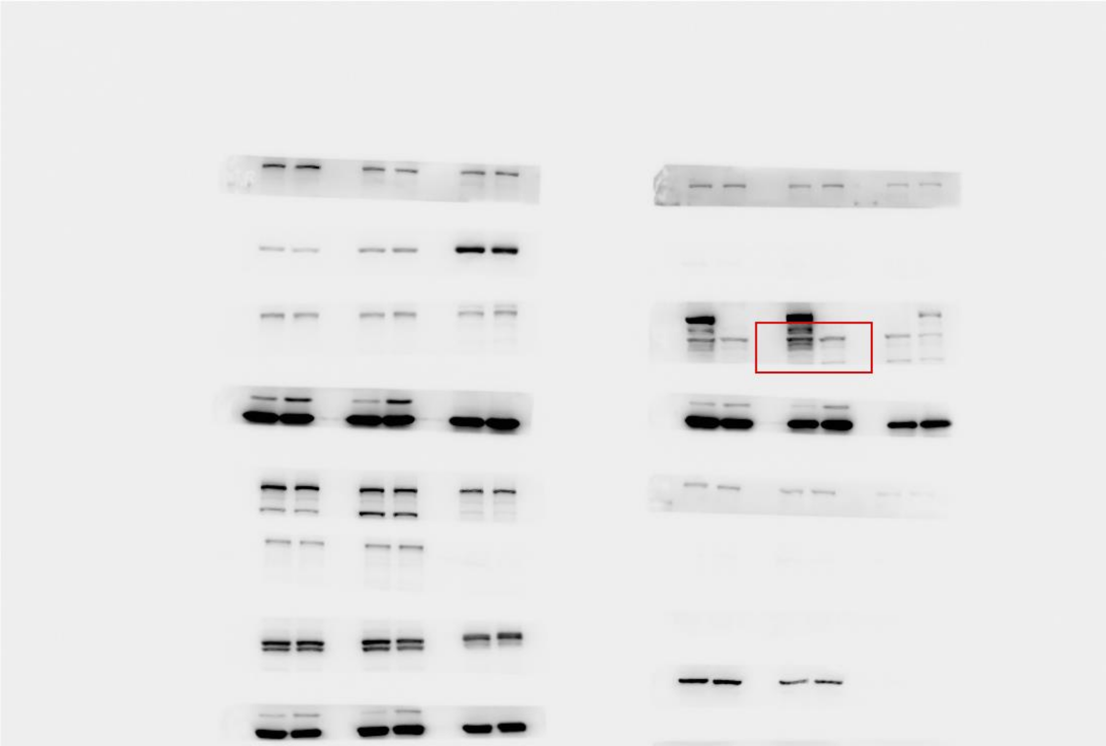

FAK

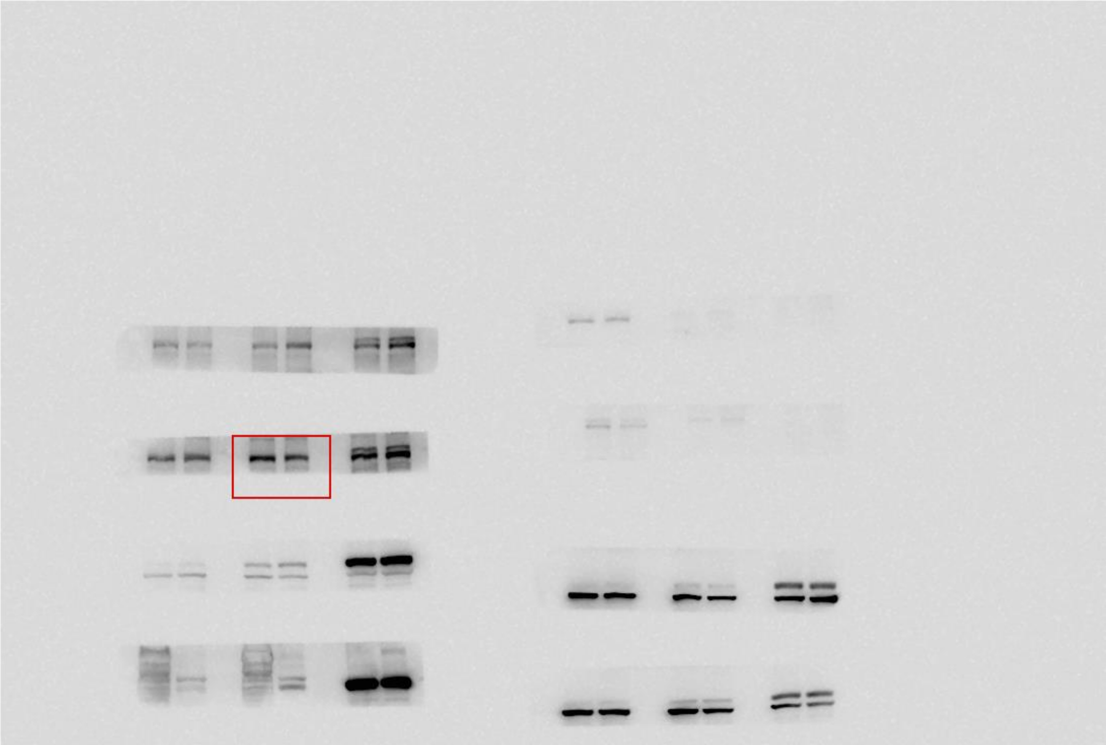

AKT

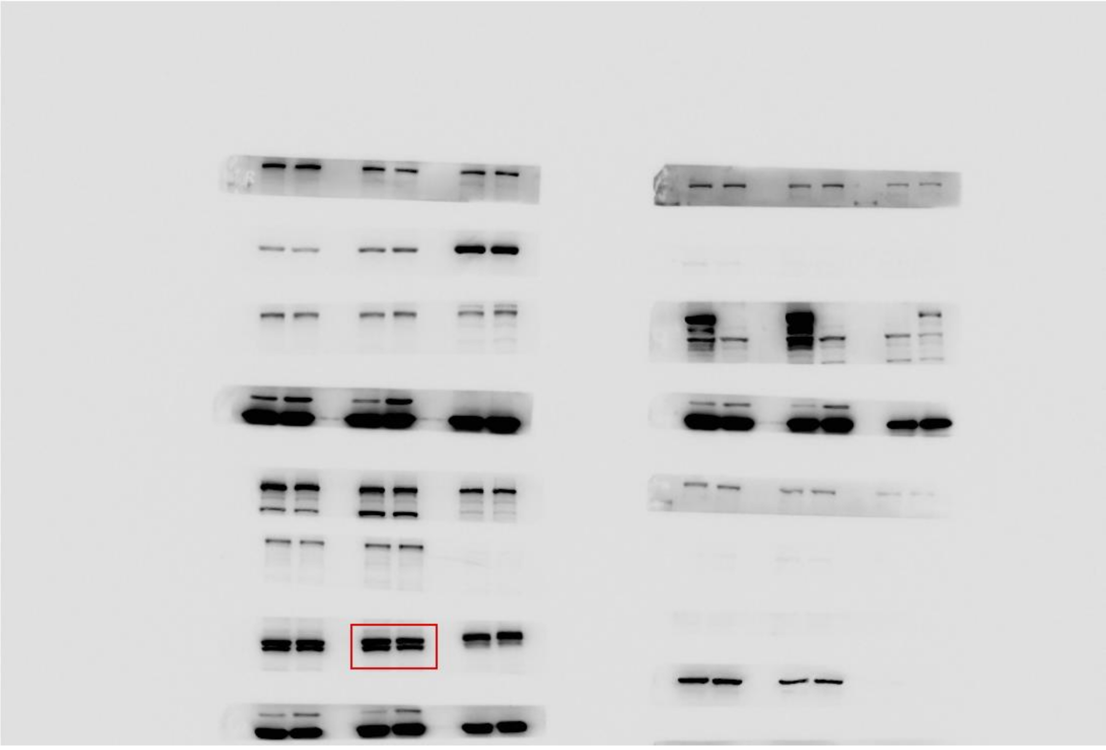

p-AKT

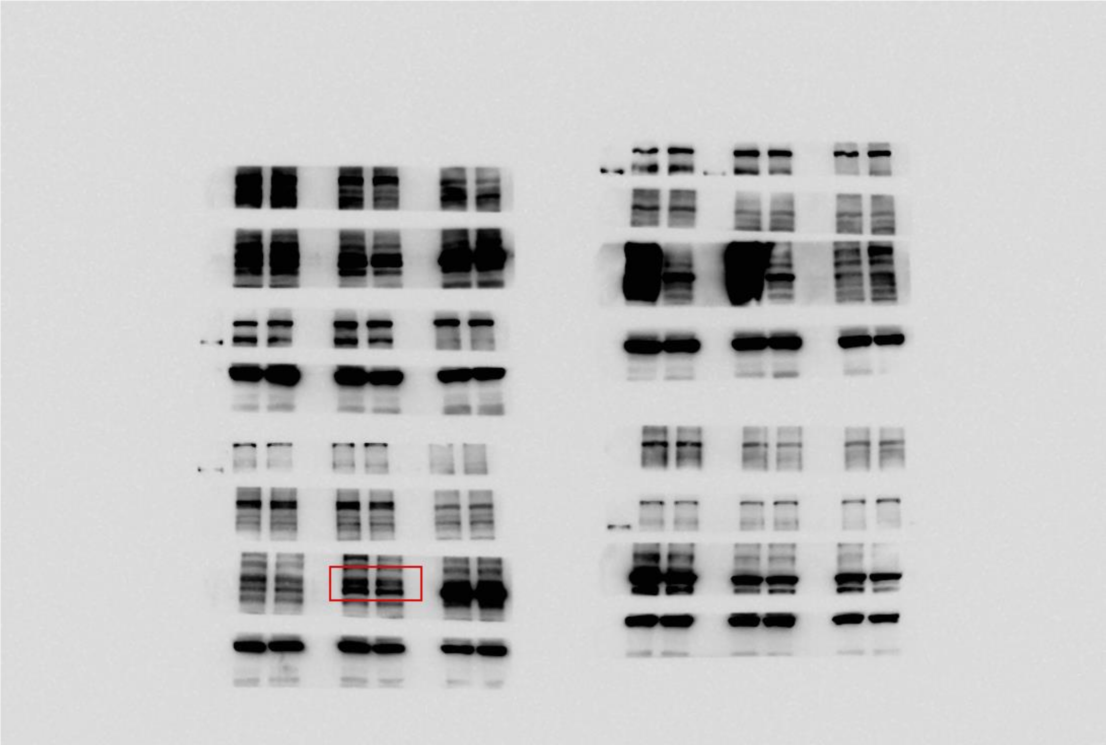

mTOR

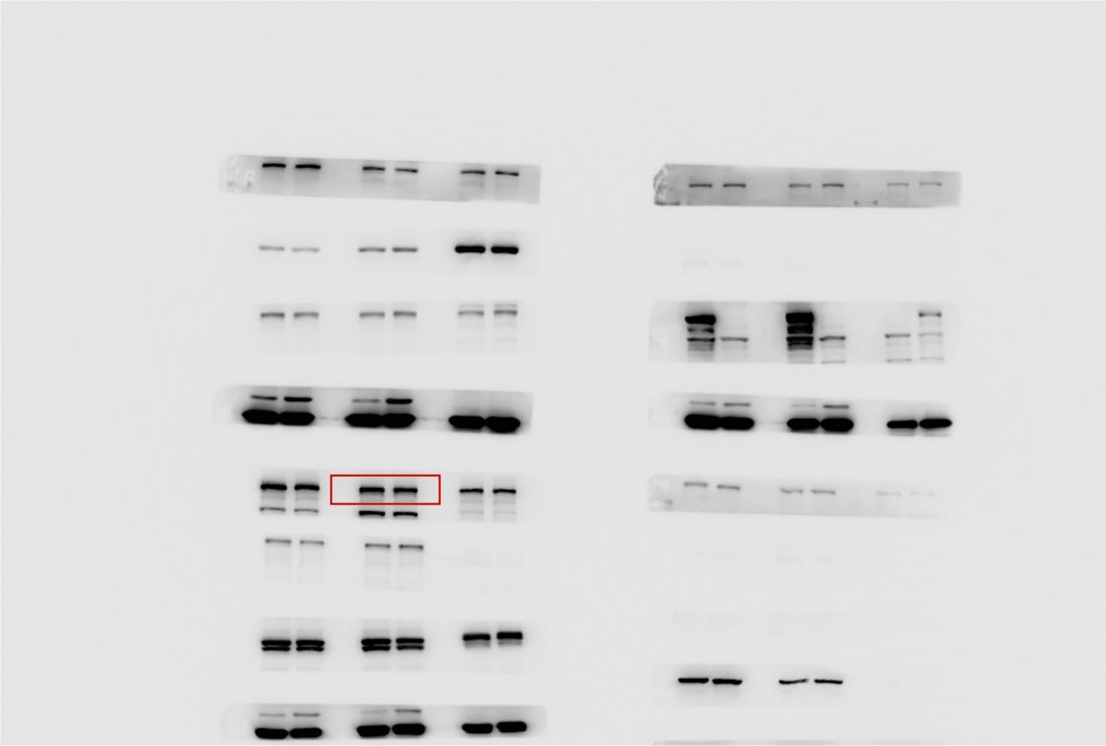

p-mTOR

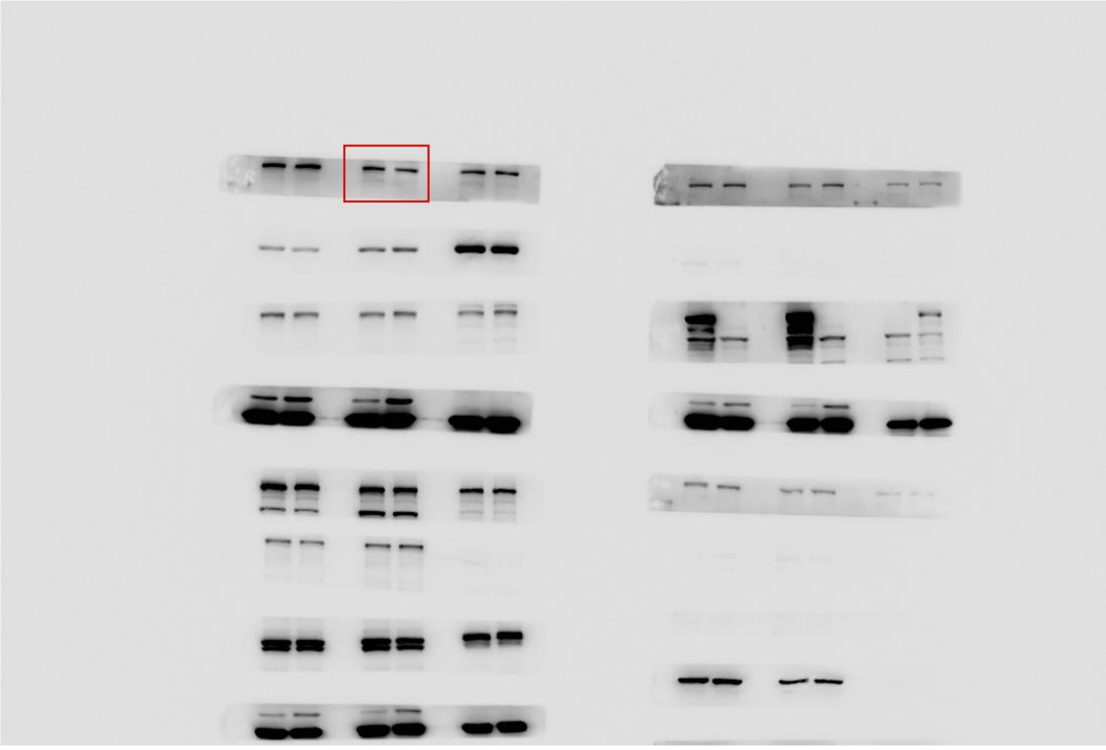

Actin

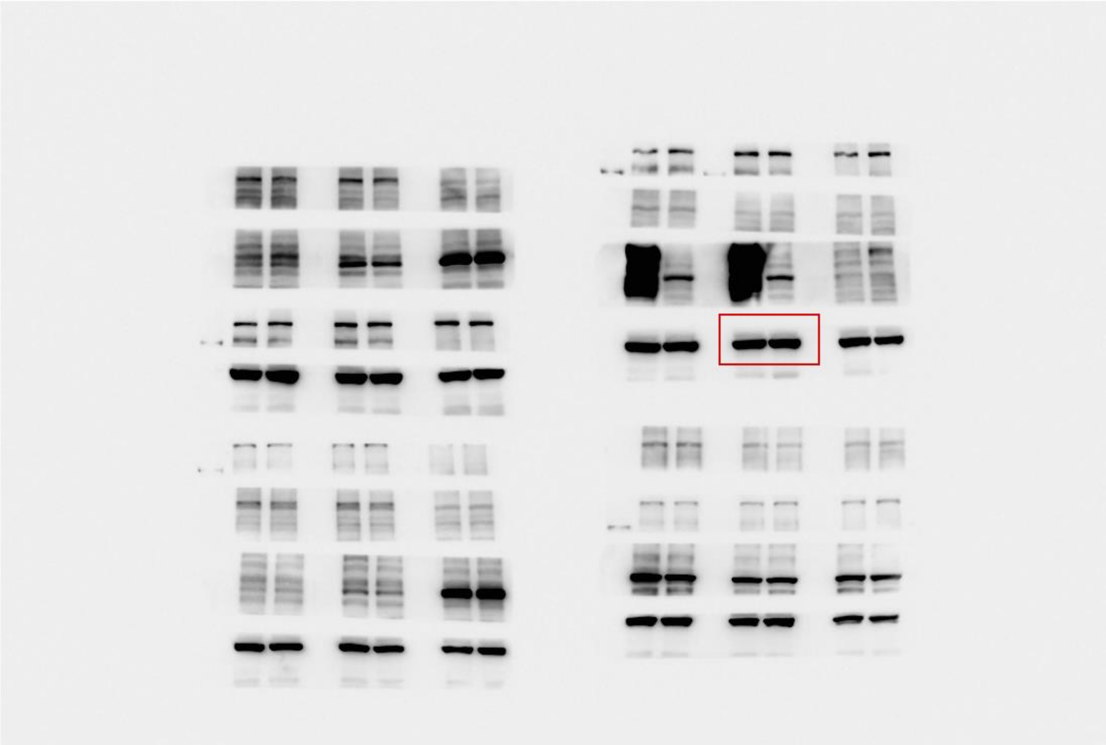

Figure 4I

LMOD1

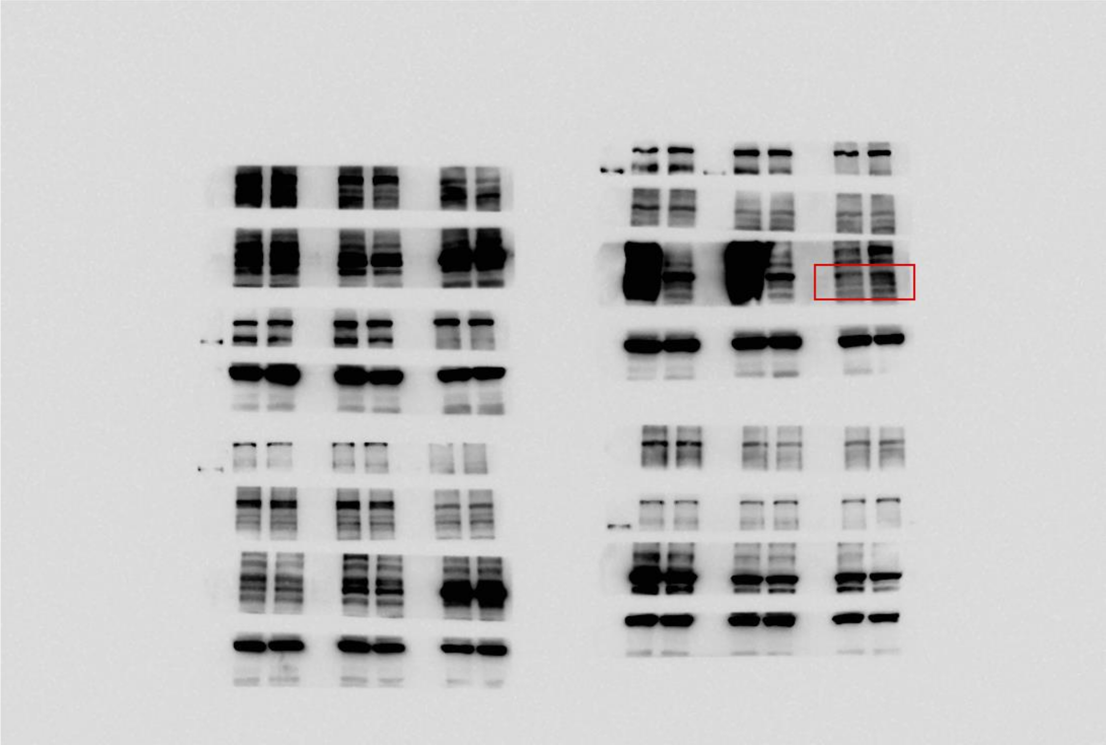

FAK

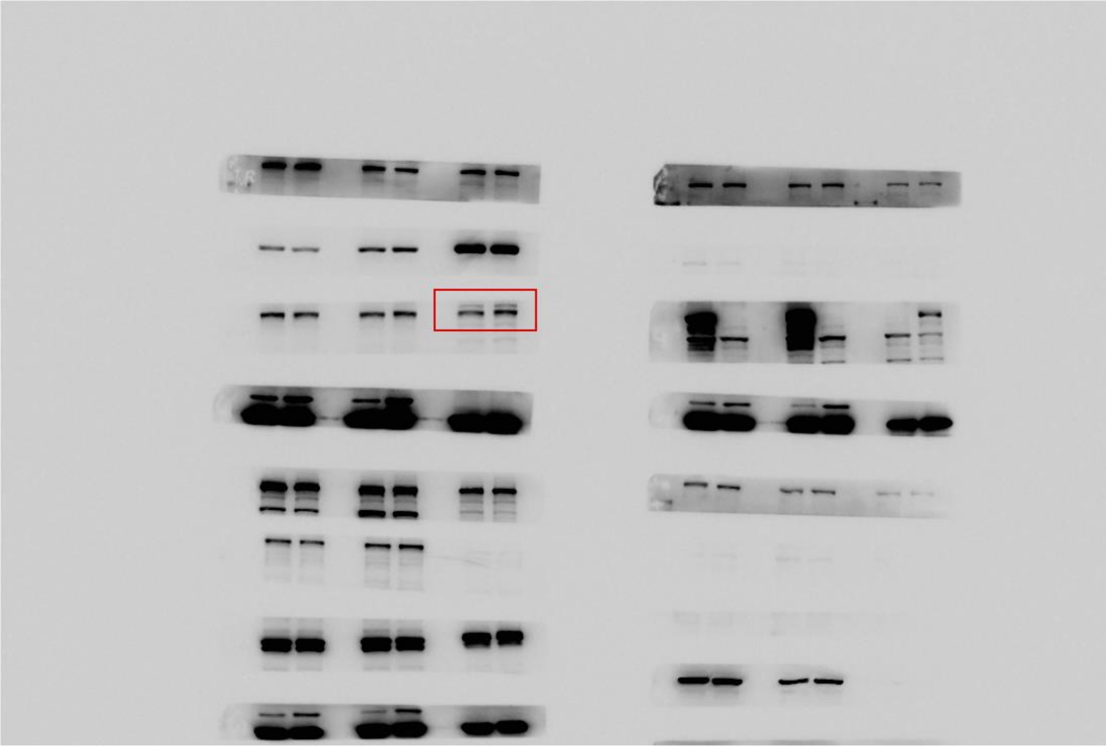

AKT

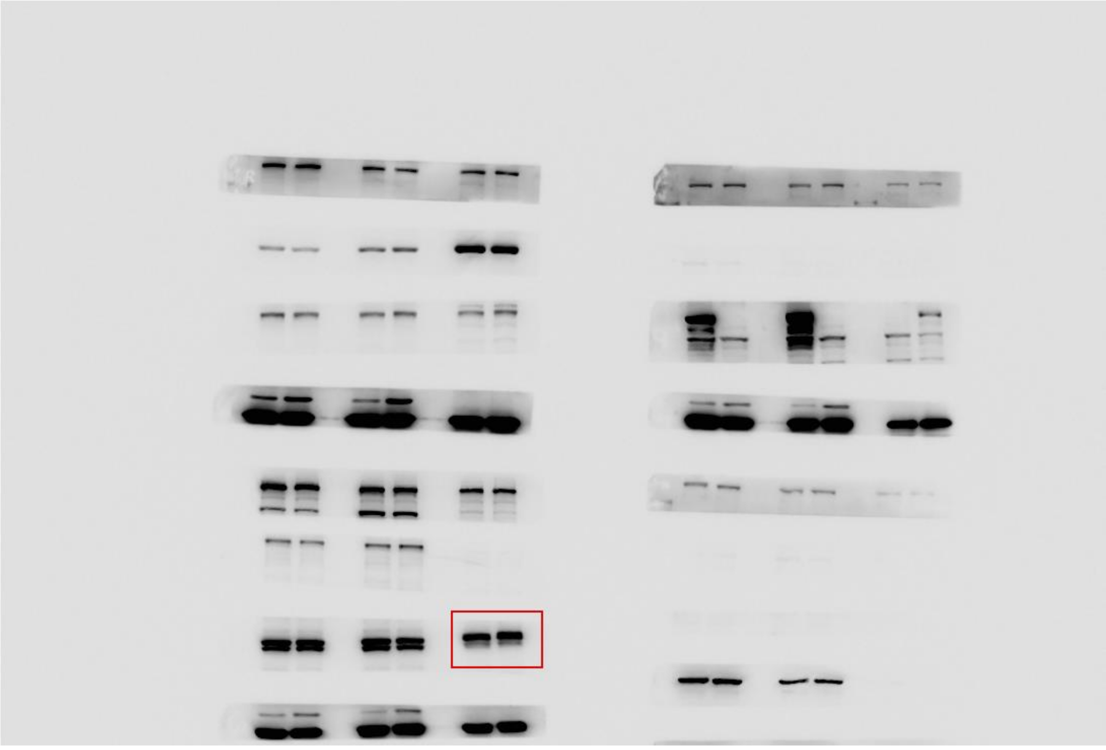

p-AKT

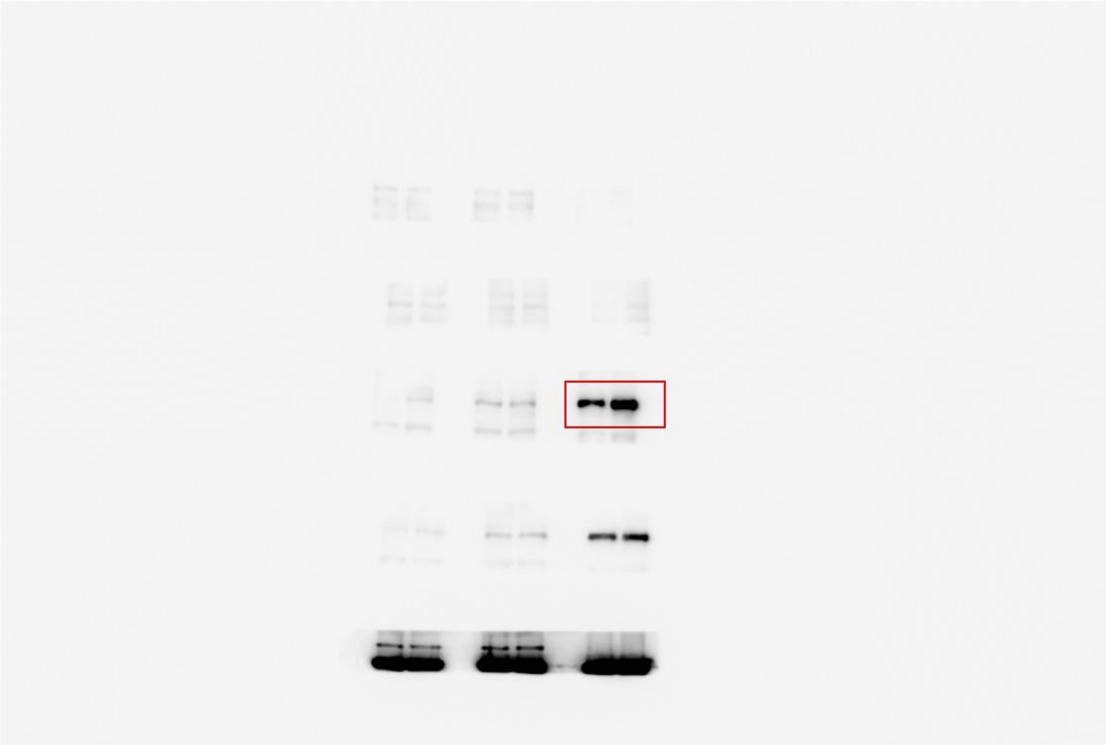

mTOR

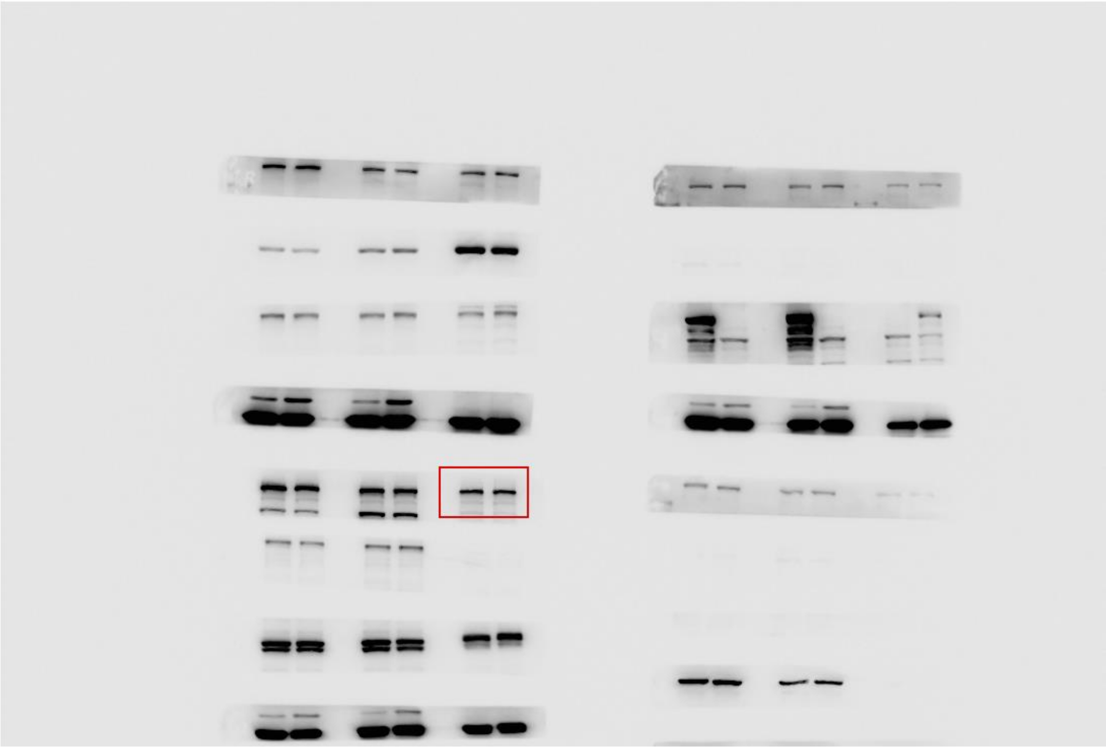

p-mTOR

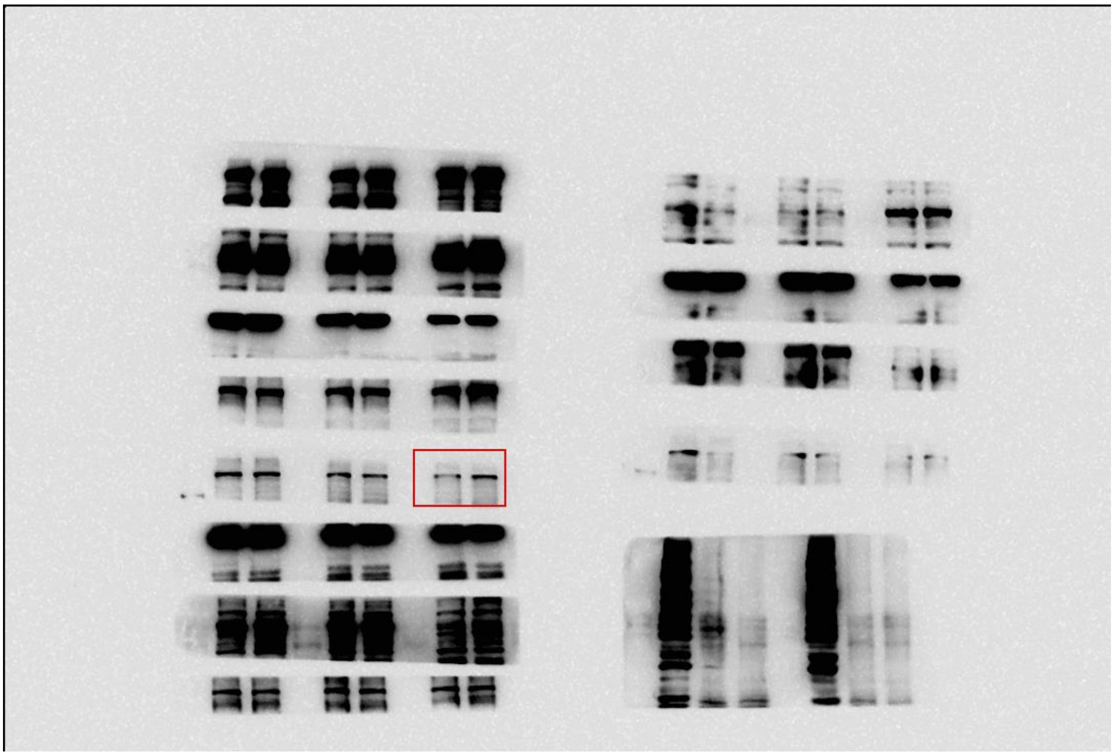

Actin

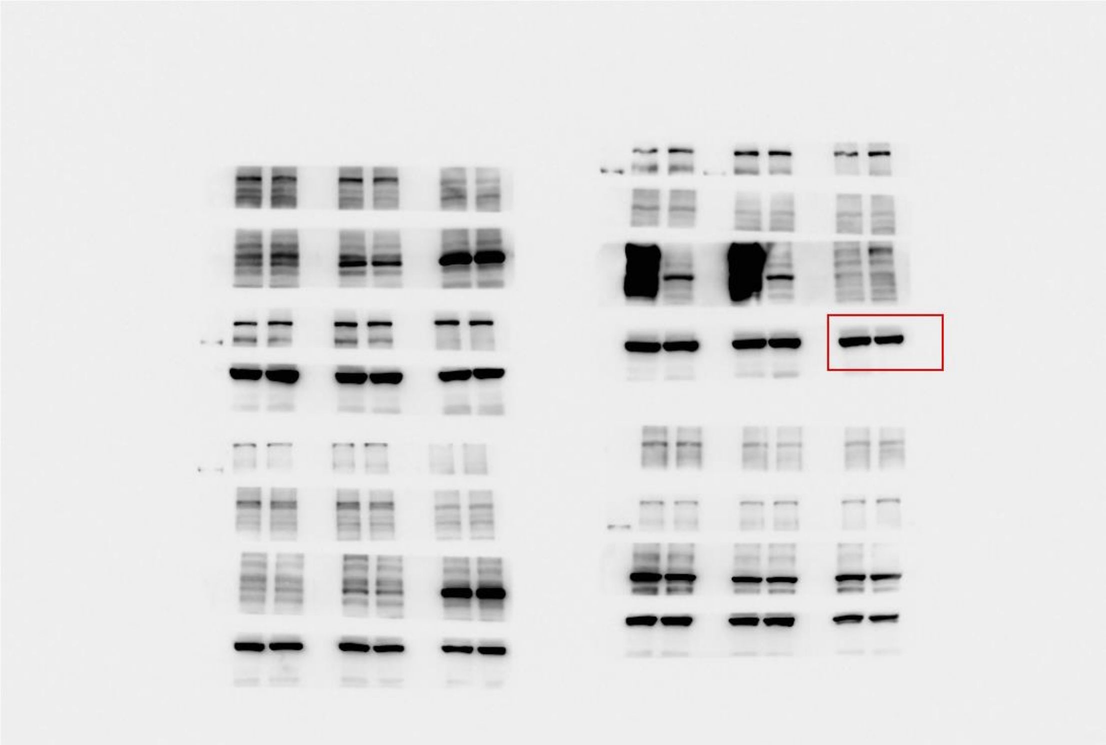

Figure 5C

E-cadherin

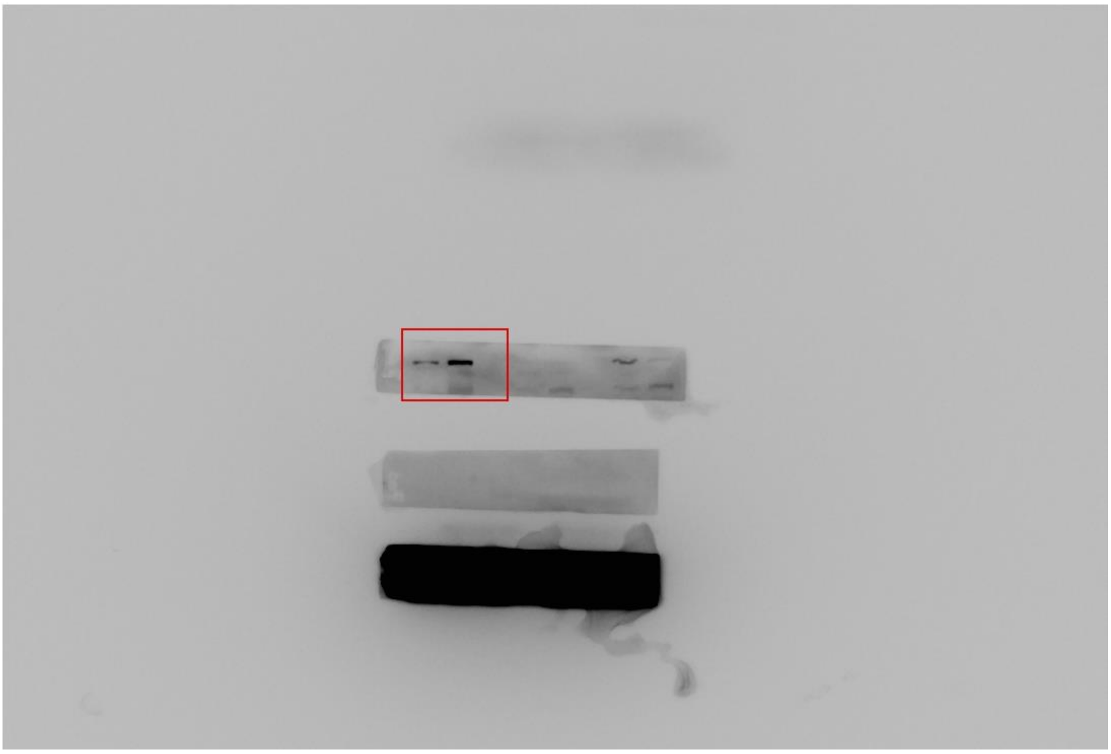

N-cadherin

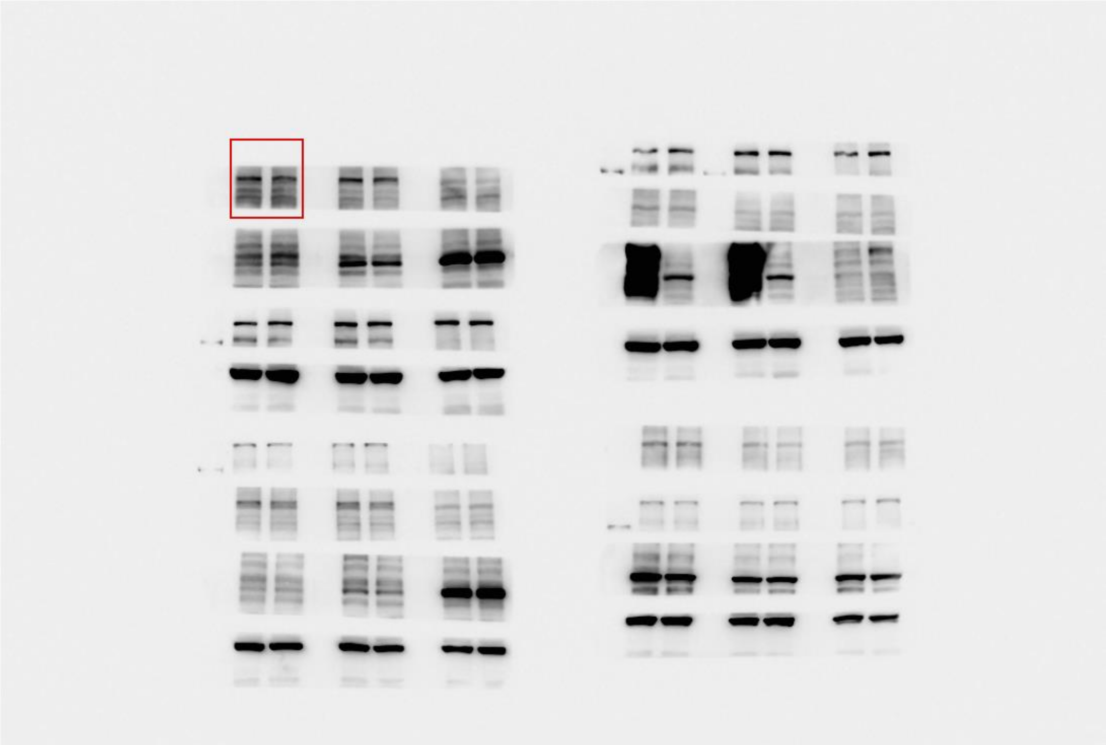

Vimentin

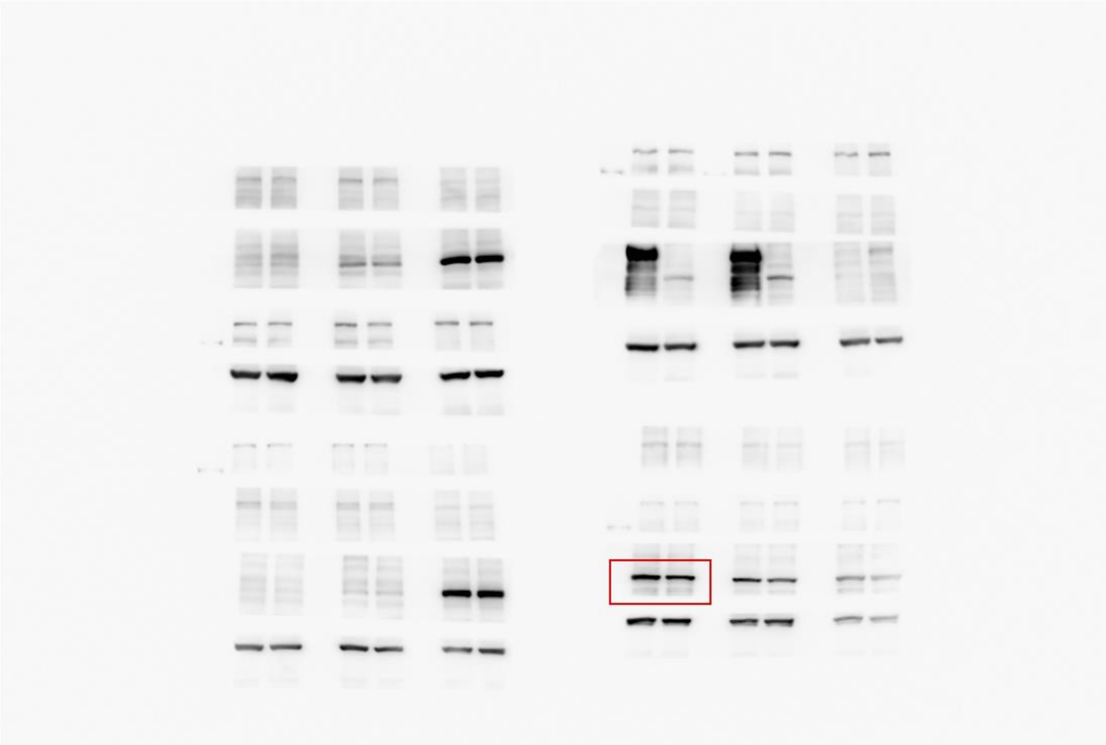

Actin

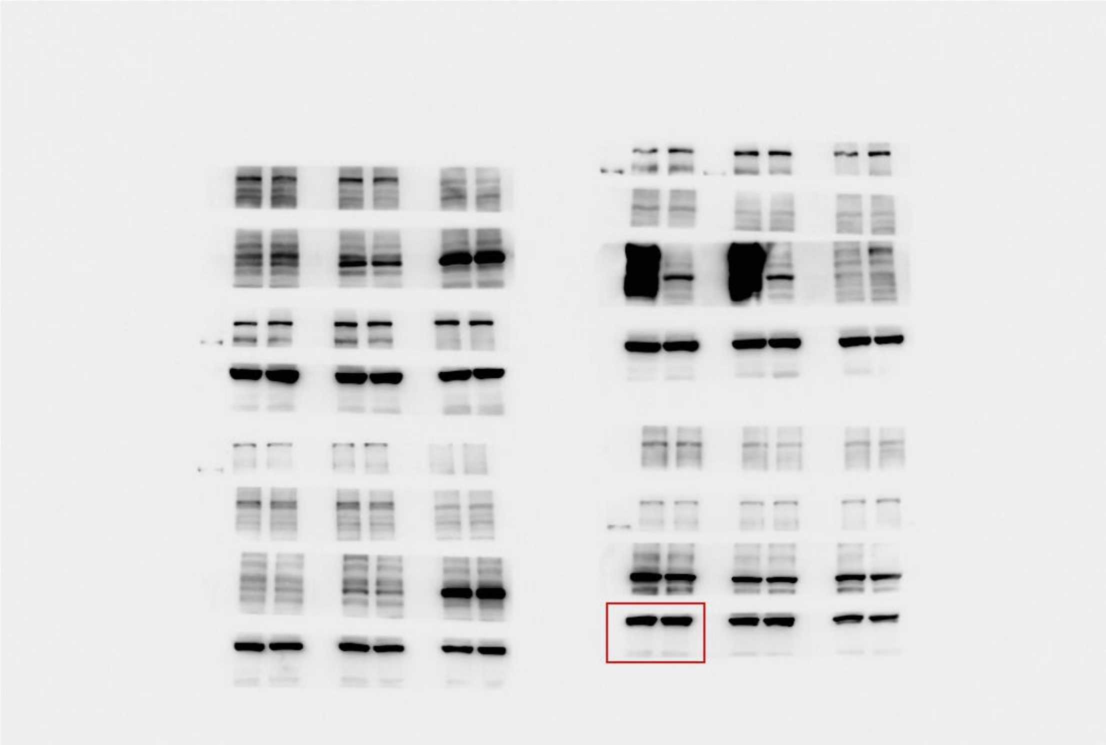

Figure 5D

E-cadherin

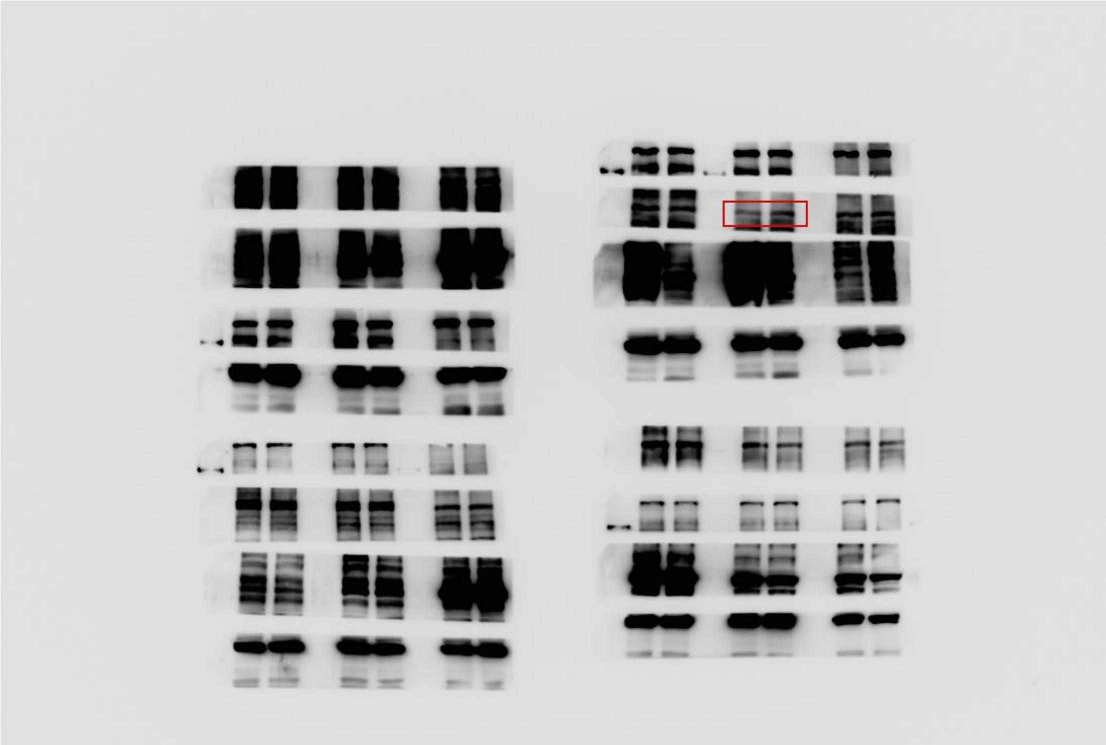

N-cadherin

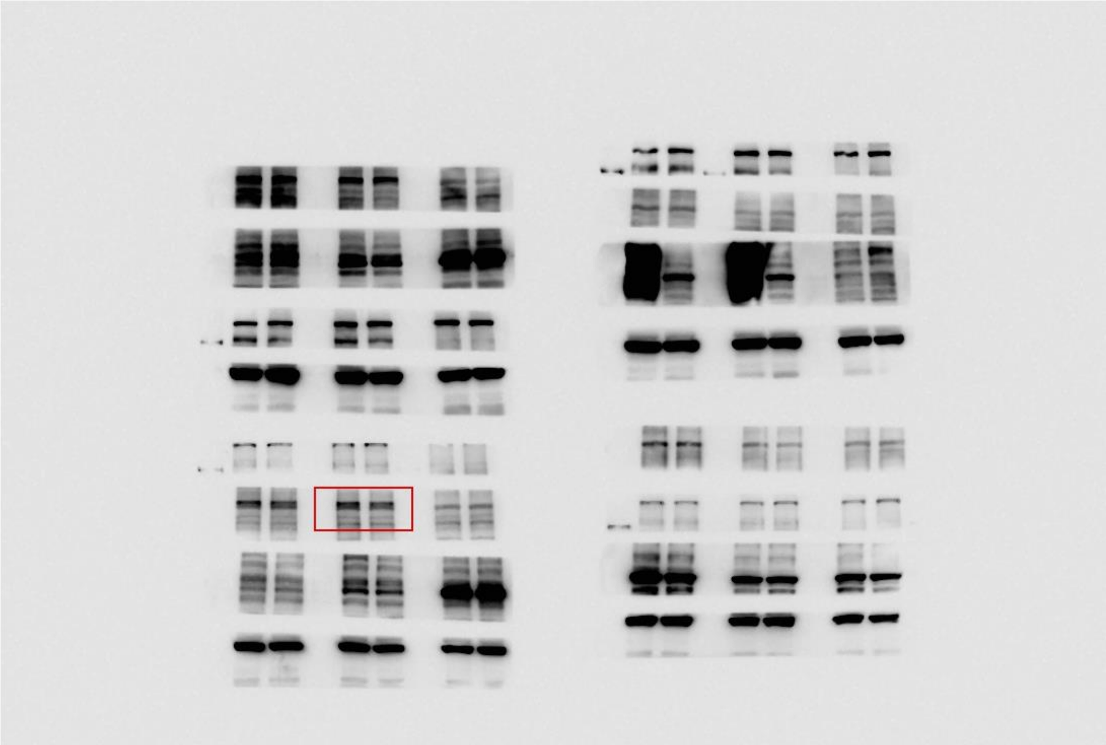

Vimentin

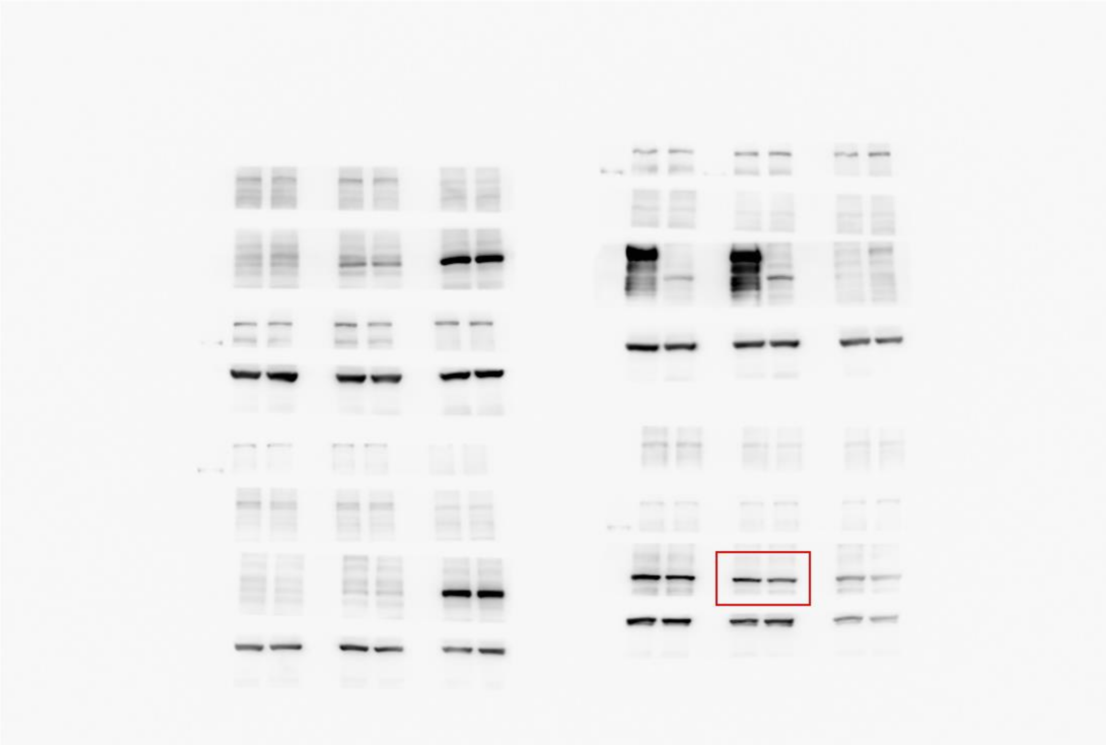

Actin

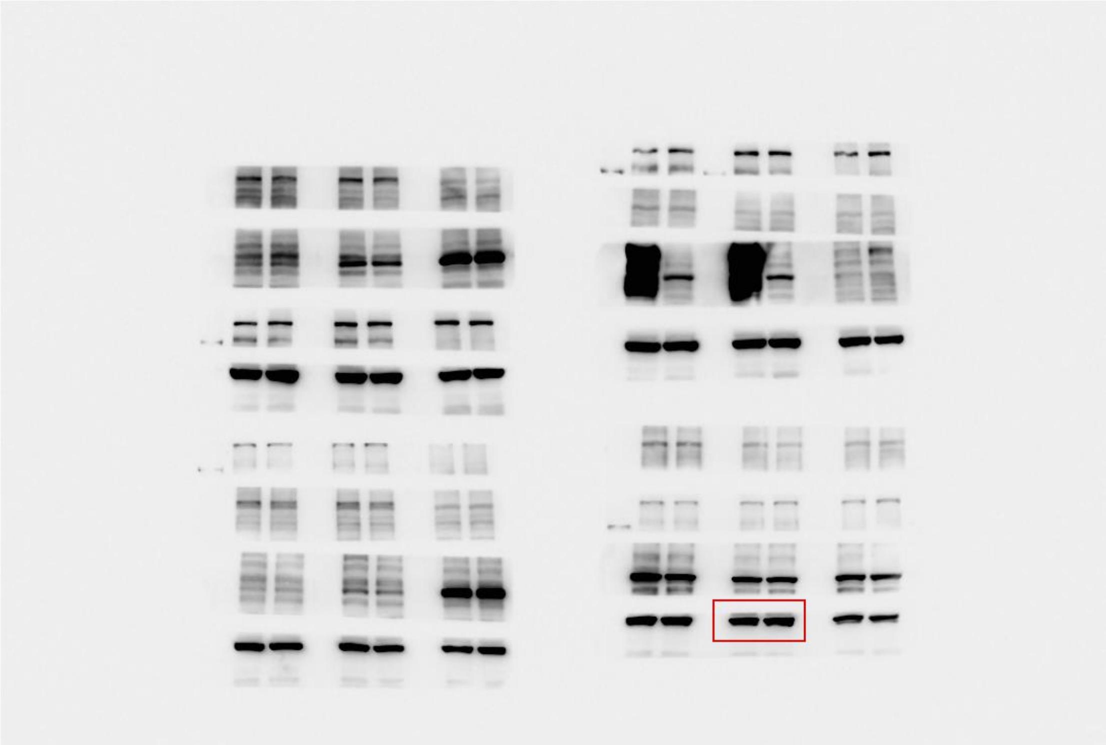

Figure 5E

E-cadherin

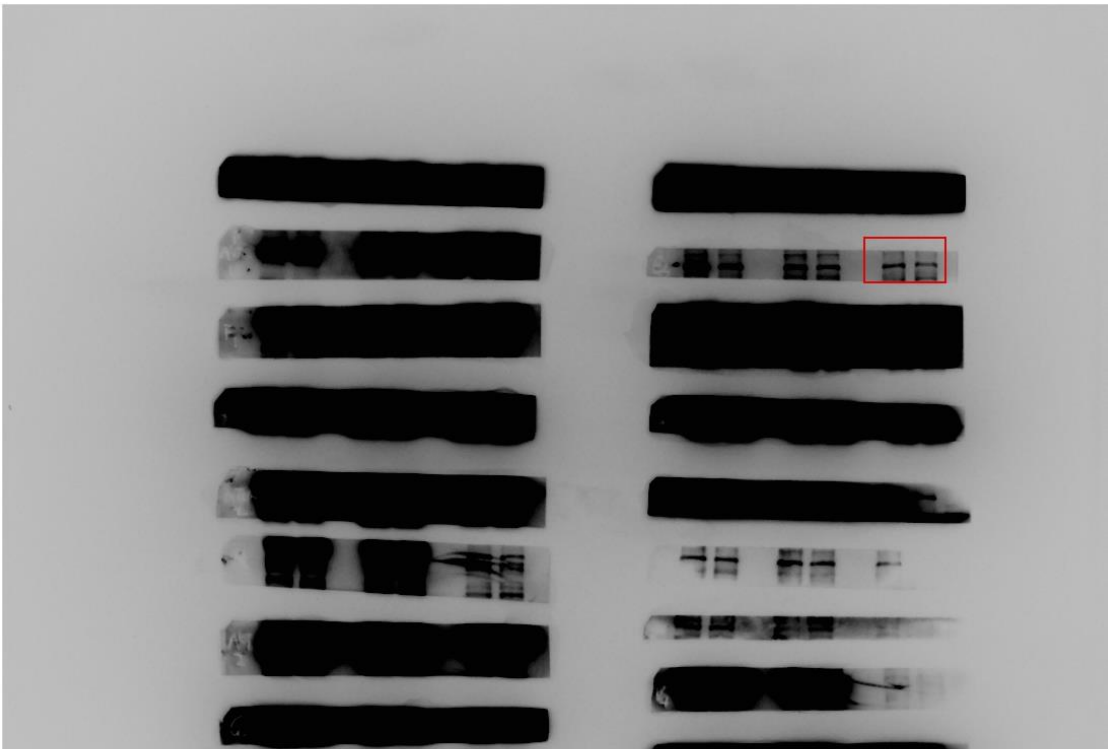

N-cadherin

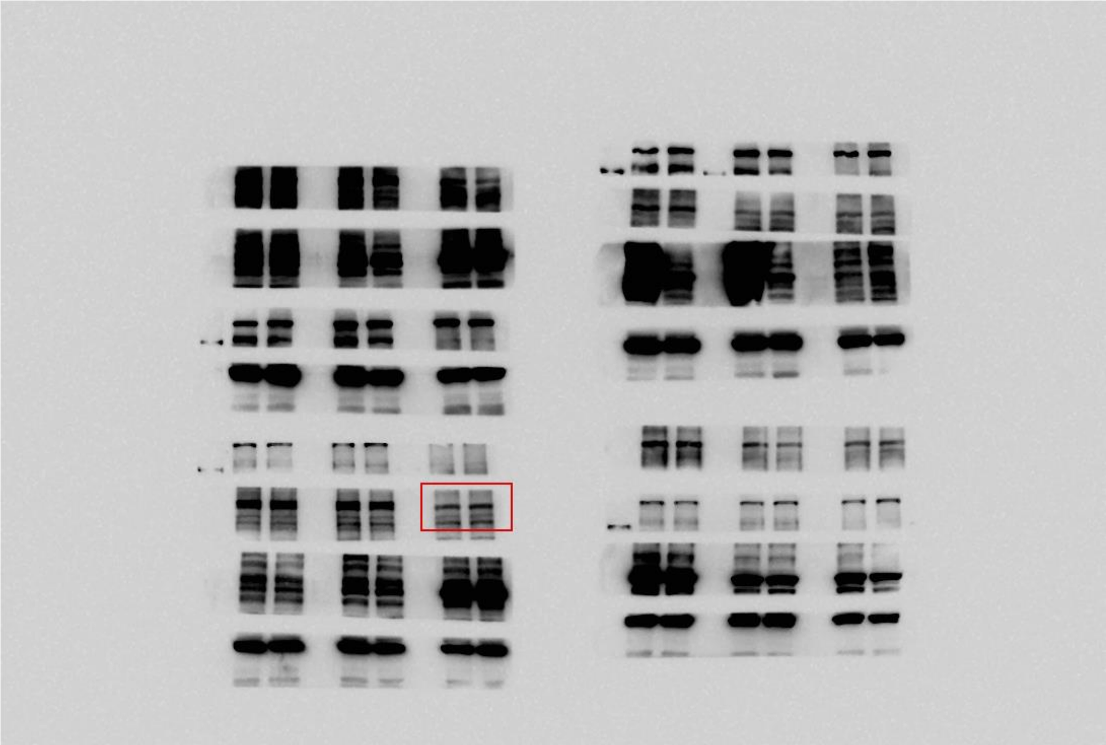

Vimentin

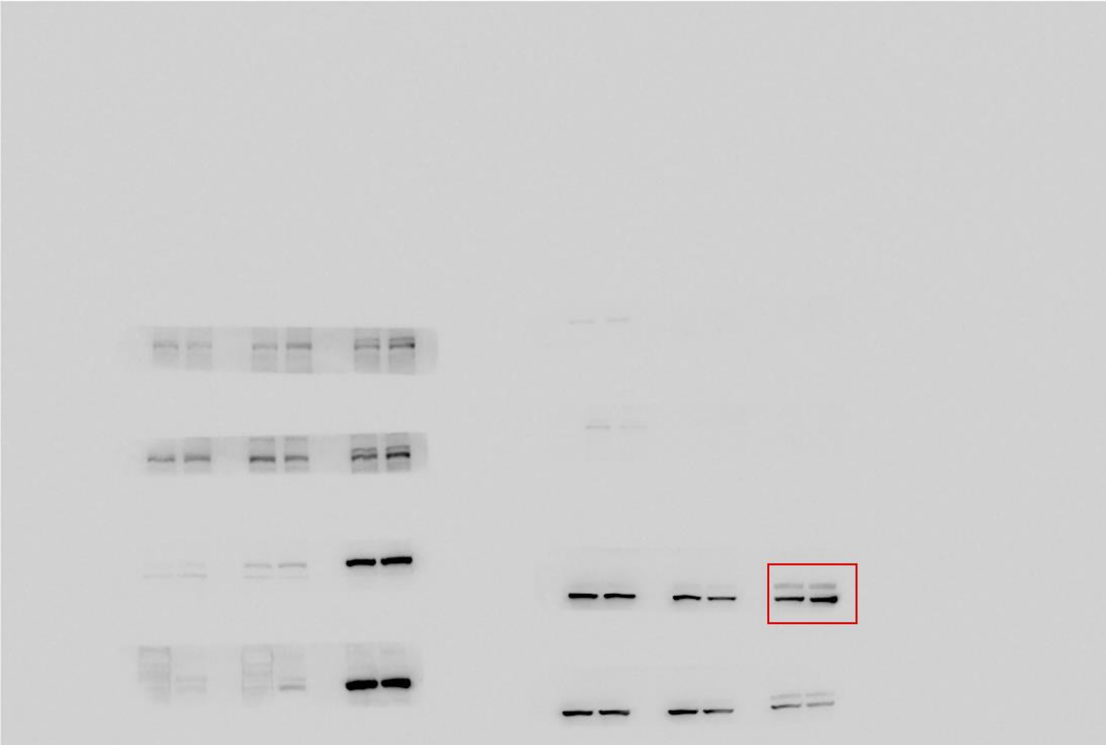

Actin

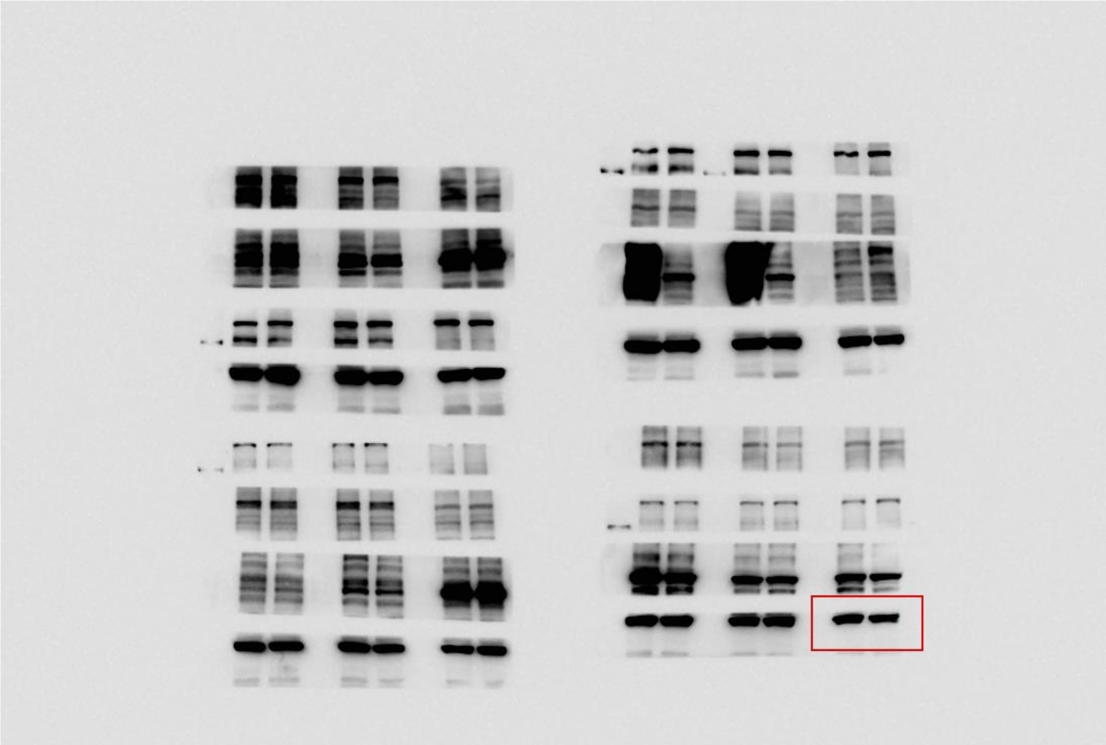

Figure 6A

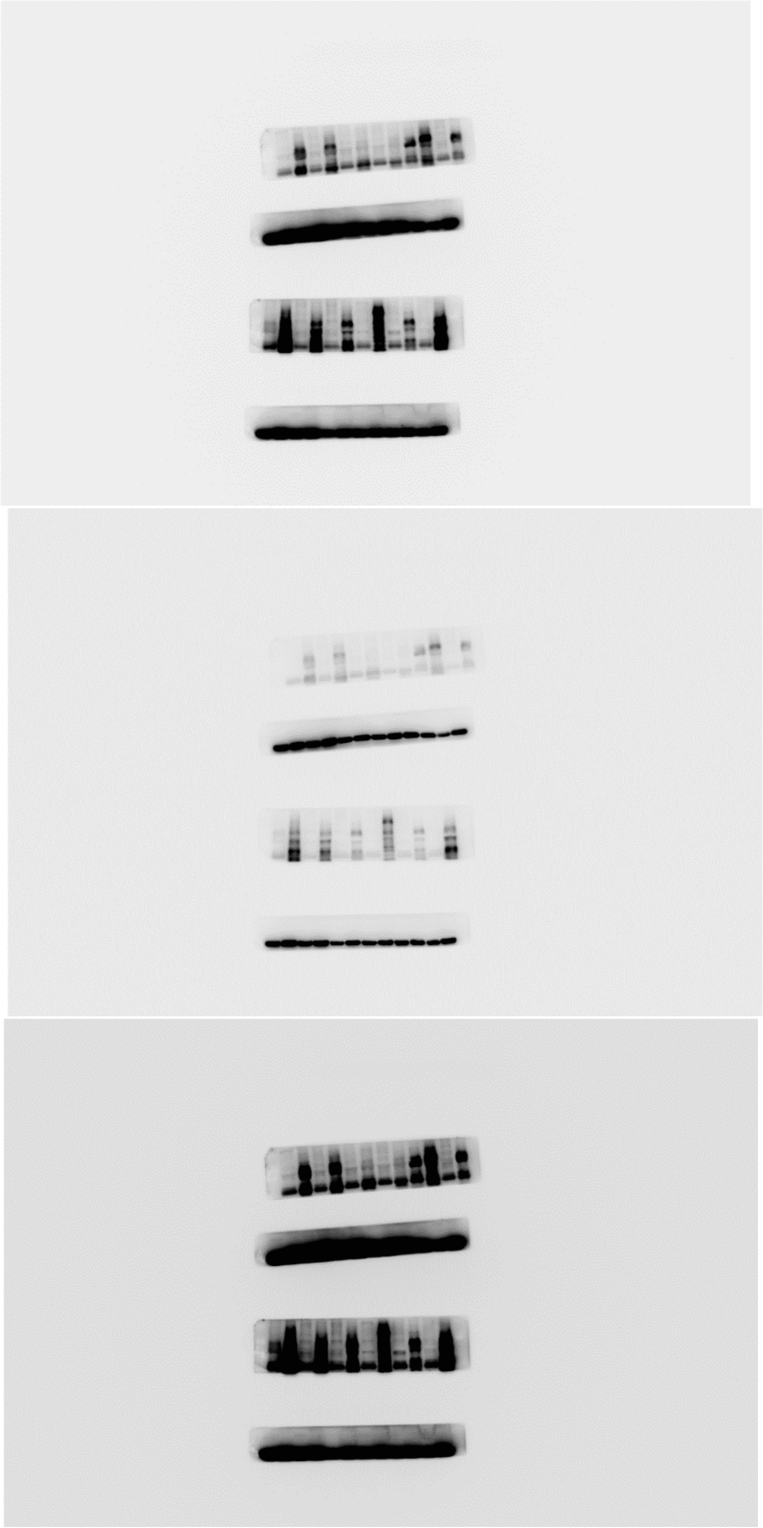

Supplement: Supplementary file 1 — Additional file 1. [file 12885_2022_9541_MOESM1_ESM.pdf]
